# A chromosome-scale assembly of the sorghum genome using nanopore sequencing and optical mapping

**DOI:** 10.1038/s41467-018-07271-1

**Published:** 2018-11-19

**Authors:** Stéphane Deschamps, Yun Zhang, Victor Llaca, Liang Ye, Abhijit Sanyal, Matthew King, Gregory May, Haining Lin

**Affiliations:** 1 Corteva Agriscience™, Agriculture Division of DowDuPont™, 8325 NW 62nd Avenue, Johnston, IA 50131 USA; 2 Corteva Agriscience™, Agriculture Division of DowDuPont™, 4010 Point Eden Way, Hayward, CA 94545 USA; 3 Corteva Agriscience™, Agriculture Division of DowDuPont™, The V-Acendas, Atria Block, 12th Floor, Plot No. 17, Hyderabad, 500081 Telangana India

## Abstract

Long-read sequencing technologies have greatly facilitated assemblies of large eukaryotic genomes. In this paper, Oxford Nanopore sequences generated on a MinION sequencer are combined with Bionano Genomics Direct Label and Stain (DLS) optical maps to generate a chromosome-scale de novo assembly of the repeat-rich *Sorghum bicolor Tx430* genome. The final assembly consists of 29 scaffolds, encompassing in most cases entire chromosome arms. It has a scaffold N_50_ of 33.28 Mbps and covers 90% of the expected genome length. A sequence accuracy of 99.85% is obtained after aligning the assembly against Illumina *Tx430* data and 99.6% of the 34,211 public gene models align to the assembly. Comparisons of *Tx430* and *BTx623* DLS maps against the public *BTx623* v3.0.1 genome assembly suggest substantial discrepancies whose origin remains to be determined. In summary, this study demonstrates that informative assemblies of complex plant genomes can be generated by combining nanopore sequencing with DLS optical maps.

## Abstract (web summary)

Assembly of large, repeat-rich eukaryotic genomes remains challenging. Here, the authors use BioNano Genomics DLS optical mapping and single-molecule nanopore sequencing to generate a chromosome-scale assembly of a new *Sorghum bicolor* accession and identify variation compared to the publicly available *S. bicolor* genome.

## Introduction

Single-molecule long-read sequencing technologies allow complex structural aspects of genomes to be elucidated in a way that shorter second-generation sequencing reads typically cannot, mainly by producing long contiguous sequences from single-molecule PCR-free DNA templates^1,2^. Two long-read sequencing technologies, from Pacific Biosciences (PacBio) and Oxford Nanopore Technologies (ONT), are commercially available. Oxford Nanopore sequencers measure changes in ionic current when the DNA fragments translocate through protein nanopores in a semi-synthetic insulated membrane. Because the detection process does not require enzyme-based nucleotide incorporation and detection of fluorescent signals, ONT sequencing read length is theoretically only limited by the length of the DNA fragment translocating through the pore. Consequently, sequences >2 Mbps in length have been reported. This read length advantage is partially offset by the fact that individual raw sequencing reads tend to have higher error rates than second-generation sequencing reads, thus requiring sequence correction and post-assembly sequence polishing to generate high-quality sequencing data. Shortly after its inception, studies have shown that the ONT technology could be used for assembling microbial genomes^3–5^. Continuous improvements in chemistry, base calling, and sequencing throughputs have recently allowed researchers to use the technology for producing high-quality whole genome assemblies for larger genomes, in combination with Illumina sequencing reads, in species such as human^6^, *Arabidopsis thaliana*^7^, and the wild tomato species *Solanum pennellii*^8^.

Numerous genomic assembly projects, including maize^9^ and *Aegilops tauschii*^10^, have generated chromosome-scale scaffolds when combining long-read PacBio sequences with complementary long-range scaffolding technologies, such as Bionano Genomics optical maps or Hi-C proximity ligation^11^. The optical mapping technology developed by Bionano Genomics has been used in genome sequencing projects to provide contig sequence validation, error correction, and high-level scaffolding resulting in significant increases in contiguity and quality^12,13^. Recently, Bionano Genomics introduced a new labeling concept, called Direct Label and Stain (DLS). In DLS, the Direct Labeling Enzyme 1 (DLE-1) attaches a single fluorophore to specific sequence motifs. Unlike previous labeling strategies, which were based on nicking endonucleases, DLS does not create damage in DNA at specific sites, thus avoiding the loss of DNA information and truncation due to fragile sites, resulting in a significant increase in continuity. Most tested complex genomes yield 50–100 times higher maps N_50_ than those generated using an endonuclease approach, often leading to the assembly of full chromosome arms or even entire chromosomes^14^.

Chromosome-scale assemblies of repeat-rich plant genomes could be used for many applications, including the study of structural variations in large regions, expected recombination frequencies in specific regions, target sequence characterization and modification for gene editing, or genomic characterization of transgenic material. In this study, the *Sorghum bicolor* inbred *Tx430* transformation line^15^ was chosen as a model plant system for whole-genome sequencing and assembly. Sorghum represents an economically important staple crop—it is the third most produced cereal crop in the US, and its adaptability to drought and high temperatures has made it a preferred crop to grow in semi-arid regions. While its genome, at ~730 Mbps, is smaller than other more complex crop genomes such as maize (~2.3 Gbps)^16^ or soybean (~1.2 Gbps)^17^, its high repeat content (~61%) and presence of large transposon structures makes it an attractive model for testing the effectiveness of the ONT chemistry for overall correctness and contiguity of a complex plant genome assembly. In addition, a reference genome has been produced via Sanger sequencing for genotype *BTx623*^18,19^, making it a valuable resource for the direct evaluation of the ONT-based assembly. In order to assess its structural integrity, the *Tx430* ONT assembly was aligned to a *Tx430* optical map and the resulting scaffolds were compared to the reference *BTx623* genome. The *Tx430* optical map was generated specifically for this study with the new DLS technology from Bionano Genomics. The overall contiguity and accuracy of the assembly was further defined through alignments of ONT contig sequences to multiple datasets, including a *Tx430* linked-read dataset generated using the 10X Genomics Chromium technology. In addition, structural discrepancies detected between the *Tx430* optical map and the reference *BTx623* genome were further confirmed by generating a second DLS optical map from *BTx623* and comparing it to the reference *BTx623* genome. Results shown here suggest that the ONT technology can be used to quickly and cost-effectively generate informative assemblies and, in combination with long-range DLS optical maps from Bionano Genomics, can generate chromosome-scale assemblies to assess the overall structural integrity of large and repeat-rich plant genomes.

## Results

### MinION sequencing metrics and characterization

High molecular weight (HMW) genomic DNA was extracted from sorghum *Tx430* adult leaf tissue using a protocol originally designed for the extraction of DNA for BAC library construction^20^. Prior to library construction, short-read DNA fragments were generated after shearing DNA to ~8 Kbps with a g-TUBE (Covaris), while long-read DNA fragments were generated after size-selecting un-sheared DNA to 20 Kbps and above with a PippinHT system (Sage Science). Libraries were prepared with the ONT Ligation Sequencing Kit 1D (SQK-LSK108), except for three long-read libraries prepared with the ONT Rapid Sequencing Kit 1D (SQK-RAD002), then sequenced for 48 h using MinION R9.4 and R9.5 flow cells. The resulting fast5 files were processed using the Albacore base caller (version 2.0.1). A total of 4.6 million reads totaling 33.5 Gbps, were generated from the short-read libraries (read length N_50_ 8633 bps) while 1.9 million reads were generated from the long-read libraries, totaling 33.5 Gbps (read length N_50_ 29,584 bps) with a maximum read length of 767,850 bps. Sequencing metrics for the flow cells used in this study are summarized in Supplementary Table 1. Assuming a 732.2 Mbps sorghum genome size, short-read and long-read sequences amounted to 91.7× equivalent of the genome. Interestingly, 68.54% of the raw short reads and 76.99% of the raw long reads had a sequence identity to the sorghum reference genome below 80%, confirming what has been observed in the previous studies^8^, and further suggesting that the Albacore sequencing pipeline software used here was not optimized for unamplified plant genome DNA.

### MinION sequence correction and assembly

Several tools, including Canu^21^, miniasm/minimap^22^, and SMARTdenovo^23^, have been developed for producing high-quality genome assemblies from single-molecule, error-prone, long reads. Analysis done internally with a sorghum short ONT read dataset corresponding to ~78× sequencing coverage equivalent of the genome had shown that Canu assembly of Canu-corrected reads yielded an initial assembly made of 8362 contigs (contig N_50_ 125,209 bps) while the same Canu-corrected reads assembled with SMARTdenovo yielded an assembly made of 3564 contigs (contig N_50_ 292,727 bps). In addition, the ONT-based genome assembly of the tomato species *S. pennellii* suggested that a combination of Canu for correction and SMARTdenovo for assembly generated the best results^8^ and Canu was described as requiring almost two orders of magnitude more CPU hours than SMARTdenovo^8^. Therefore, SMARTdenovo was selected for the final de novo assembly of the Canu-corrected sorghum ONT reads.

Prior to the final assembly, the effects of ONT read depth and length on the assembly were assessed. In all cases, only reads larger than 2 Kbps in length were submitted to Canu correction while only Canu-corrected reads larger than 5 Kbps were submitted to SMARTdenovo assemblies. Correction and assembly were performed on subsets of ONT reads representing a 40-fold coverage equivalent of the genome, a 60-fold coverage equivalent of the genome and “long” ONT read only corresponding to ~29× coverage equivalent of the genome after Canu correction. Results (Supplementary Table 2) indicated that, expectedly, increased coverage and increased read length both led to higher contiguity of the assembly, and that low (~30×) coverage sequencing of a complex plant genome should mainly focus on sequencing long DNA fragments (>20 Kbps) for a contiguous assembly. For the final assembly, all raw reads were combined, and filtered based on length for correction and assembly as described above (Supplementary Table 3). After assembly, ONT contigs were polished twice with Pilon^24^, using an Illumina *Tx430* whole-genome shotgun dataset produced from a paired-end library with an average fragment size of ~400 bps. A total of 691.64 MM Illumina 150 bps reads were produced, totaling 103.7 Gbps, or a 141-fold coverage of the sorghum genome. Results from the assembly are summarized in Table 1.

**Table 1:** Summary of ONT assembly metrics

	Canu + SMARTdenovo	Canu + SMARTdenovo + Pilon (1×)	Canu + SMARTdenovo + Pilon (2×)
Number of contigs	734	724	723
Total length	660,557,552	671,283,418	671,866,842
Average contig length	899,942	927,187	929,276
Minimum contig length	8556	8623	8655
Maximum contig length	16,114,705	16,329,467	16,337,099
N_25_ contig length	7,217,337	7,344,315	7,351,898
N_50_ contig length	3,005,621	3,051,816	3,053,182
N_75_ contig length	1,248,066	1,261,559	1,254,124

Assembly metrics are shown before polishing with Pilon, after one round of polishing with Pilon (1×), and after two rounds of polishing with Pilon (2×). Contigs generated after two rounds of polishing were used for subsequent analysis. (Unit = bps)

As shown, the *Tx430* genome assembly produced a total of 734 contigs, with a contig N_50_ of ~3 Mbps and a mean contig length of 899.9 Kbps. Interestingly, the N_50_ contig length, but not the number of contigs, increased significantly when compared to the LR assembly (Supplementary Table 2). Two rounds of Pilon polishing with the Illumina *Tx430* whole-genome shotgun dataset slightly decreased the number of contigs to 723, while increasing the size of the assembly to ~671.8 Mbps (Table 1). Distribution of contig lengths (see Supplementary Figure 1) indicates that 97% of the assembly, or 651.3 Mbps, was contained within the largest 400 contigs.

An initial estimation of the accuracy of the *Tx430* ONT contig sequences was performed by mapping the Illumina *Tx430* whole genome shotgun data against the ONT contigs, using Bowtie 2^25^. After alignment, ONT sequence accuracy was determined against the uniquely aligned Illumina reads. Results showed that 92.94% of uniquely aligned Illumina reads (corresponding to 229.88 MM reads) exhibited >99% sequence identity (including 84.95% with 100% sequence identity) to the ONT assembly while only 81.03% of the reads had >99% sequence identity (64.78% with 100% sequence identity) to the public v3.0.1 assembly. After two rounds of Pilon polishing, the average accuracy rate of all ONT contigs to the uniquely aligned Illumina reads was 99.62%. Interestingly, additional polishing of the ONT contig sequences with nanopolish^3^, followed by two additional rounds of Pilon polishing, only slightly increased the accuracy to the uniquely aligned Illumina reads to 99.67%, while decreasing the alignment rate of Illumina data from 97.74 to 96.84%.

**Fig. 1:**
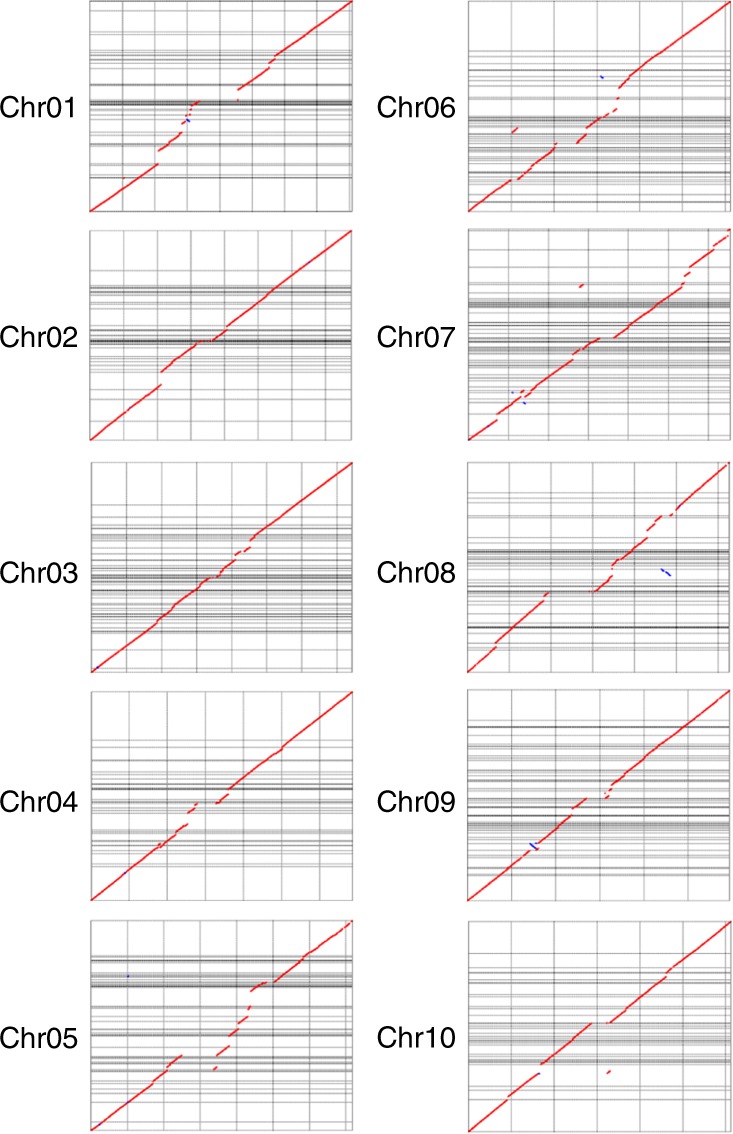
MUMmerplot comparison of *Tx430* ONT assembly with the *BTx623* reference assembly. ONT contigs (*Y*-axis) (*Tx430*) were aligned to all 10 chromosomes from the public *BTx623* v3.0.1 genome assembly (*X*-axis) (*BTx623)* with NUCmer, and results were subsequently filtered for 1-on-1 alignments and rearrangements with a 20 Kbps length cutoff. *BTx623* chromosomes are labeled by number and contain multiple megabase-scale regions in the form of unresolved nucleotide sequences

To test the completeness of the *Tx430* ONT assembly, contig sequences were compared to individual chromosome sequences from the public v3.0.1 *S. bicolor* genome assembly. NUCmer v3.1 (MUMmer 3.23 package)^26^ generated a total of 2376 partial contig alignments with sequence identity to the reference averaging 97.67% and ranging from 95.02 to 100%. Comparisons were made using MUMmerplot 3.5 (MUMmer 3.23 package) on the NUCmer alignments, which were subsequently filtered for 1-on-1 alignments and rearrangements with a 20 Kbps length cutoff (Fig. 1). Despite the incomplete nature of the v3.0.1 *S. bicolor* assembly in its pericentromeric regions, the results suggested sufficient sequence homology to the ONT contig assembly.

### Generation of a *Tx430* Bionano DLS contig map

To generate chromosome-level maps in *Tx430*, a Bionano Saphyr system was used in combination with the DLS technology. To construct optical maps in *Tx430*, a total of 1,224,604 DNA fragments with lengths ranging from 150 to 2706 Kbps and a N_50_ length of 286 Kbps were imaged and digitized (Supplementary Table 4). The final de novo assembly yielded 79 maps, with a total combined length of 732.1 Mbps. The resulting map N_50_ was 33.77 Mbps, with the largest map being 47.64 Mbps. Out of 79 maps, 32 maps accounted for 99.5% of the total assembly aligned to the *BTx623* reference (Supplementary Figure 2).

**Fig. 2:**
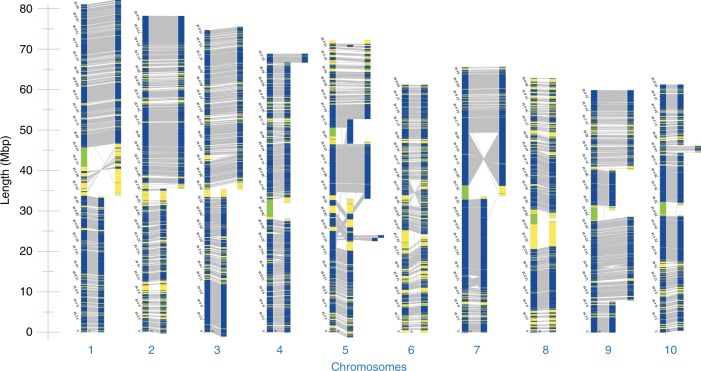
Alignment of *Tx430* maps against in-silico maps of the *BTx623* reference assembly. Collinear DLE-1 markers on the two maps are linked (gray lines). All but three chromosomes are captured by two DLS maps. Large inversions are shown on chromosomes 6 and 7. Regions in green are stretches of random nucleotides in the reference assembly. Regions in yellow exhibit breaks in collinearity between the two maps

The DLS optical maps were aligned to an in-silico map created from the reference *S. bicolor* genome assembly to determine potential structural discrepancies between the *Tx430* and *BTx623* genomes. With DLS, the majority of map breakages in plant inbred projects corresponds to long centromeric, ribosomal DNA, and segmental duplication regions, as well as regions including residual structural heterozygosity. Results are summarized in Fig. 2. In the end, all but three chromosomes were captured by only two maps, each one roughly corresponding to a chromosome arm. Only chromosome 5, 9, and 10 provided more complexity and included 7, 5, and 4 maps, respectively. The alignments suggest the presence of substantial genomic rearrangements between the two maps. For example, a large pericentromeric inversion, in relation to the optical map mapping to the same region, can be detected on chromosome 7 of *BTx623* (Fig. 2). Another large inversion is detected on chromosome 6 (Fig. 2). Additional rearrangements (Supplementary Table 4) include more than 3000 insertion/deletion events and 28 translocations.

One small 756 Kbps DLS map, located towards one end of chromosome 5, was detected overlapping with a larger 19.5 Mbps DLS map (Fig. 2). Subsequent alignments to ONT contigs showed that the 756 Kbps DLS map contained potentially unmatched DLE-1 motifs (Supplementary Figure 3). Multiple adult plants were used as source material for DNA extraction. Therefore, unmatched DLE-1 motifs could be related to residual structural heterozygosity in that region of the *Tx430* DLS optical map.

### Generation of a *Tx430* chromosome-scale hybrid assembly

To further improve the assembly, the 723 contigs were scaffolded with the sorghum *Tx430* DLS contig maps. The resulting hybrid scaffolds showed a significant improvement in contiguity when compared to the ONT contig assembly alone. The initial hybrid map assembly was made of 30 scaffolds, totaling 661.06 Mbps, or 98.4% of the original ONT assembly, with a resulting N_50_ increasing from 3 to 33.35 Mbps (Table 2).

**Table 2:** Summary of DLS optical map assembly and merging with ONT contigs

	Original DLS genome map	Original ONT contigs	ONT contigs in hybrid scaffold	ONT contigs not in hybrid scaffold	Hybrid scaffolds	Hybrid scaffolds after correction
Number of contigs/scaffolds	79	723	500	363	30	29
Total length (Mbps)	719.339	671.867	644.44	25.117	661.06	661.16
Minimum length (Mbps)	0.225	0.009	0.058	0.00006	0.086	0.115
Maximum length (Mbps)	47.659	16.337	16.337	1.549	52.621	46.577
N_50_ length (Mbps)	33.773	3.053	2.991	0.13	33.35	33.28

Merging of DLS optical maps with ONT contigs produced a hybrid assembly made of 29 scaffolds, totaling 661.16 Mbps and with a scaffold N_50_ value of 33.28 Mbps

A total of 141 conflicts in 83 ONT contigs were corrected during the hybrid map assembly process. Bionano corrections were checked by way of NUCmer v3.1 alignments of corrected ONT contigs to the v3.0.1 assembly and subsequent filtering for 1-on-1 alignments. During the scaffolding process, priority was given to corrections made by the Bionano software over ONT contig assembly data due to the length and nature of the Bionano individual molecules. MUMmerplot comparisons for seven ONT contigs (Supplementary Figure 4) confirmed their alignments to distinct v3.0.1 chromosomes, in agreement with the correction identified by the Bionano software for those same contigs, further confirming that the Bionano software correction process was accurate. Additional inspection indicated that the largest 52.62 Mbps hybrid scaffold was chimeric, generated by merging two optical maps aligning to two different chromosomes from the v3.0.1 assembly, with a small ~40 Kbps ONT contig sequence region with low DLE-1 marker count (Supplementary Figure 5). This 40 Kb region was subsequently deleted, before regenerating hybrid scaffolds with the same *Tx430* contig maps. The resulting hybrid map assembly was made of 29 scaffolds, totaling ~661.16 Mbps, with a slightly lower N_50_ length of 33.28 Mbps (Table 2).

An example of a hybrid scaffold (Supplementary Figure 6) showed ONT contigs ordered and oriented after mapping to a single DLE optical map on chromosome 6. Apparent sequence gaps in the hybrid scaffolds, represented by strings of N’s in the hybrid assembly, may correspond to genomic regions lacking a sufficient number of DLE-1 labels to merge small ONT contigs.

Out of the 723 ONT contigs used to generate the hybrid scaffolds, 363 were not included, totaling approximately 25 Mbps (Table 2). BLAST analysis showed that 26 mapped to mitochondrial sequences and 97 mapped to plant pathogens. 208 out of the remaining 240 contigs showed best hits to sorghum sequences and a RepeatMasker analysis^27^ of the 240 contigs indicated that 57.43% of their sequences were masked (including 51.88% matching with LTR elements present in Arabidopsis, rice, sorghum, or maize). A separate tandem repeat analysis of the same 240 contigs with Tandem Repeat Finder (http://tandem.bu.edu/trf/trf409.linux64.download.html) shows that an additional 29.47% of sequences were masked (including two tandem repeats containing 3913 and 2403 copy numbers, respectively, of a 7 bp motif).

**Fig. 3:**
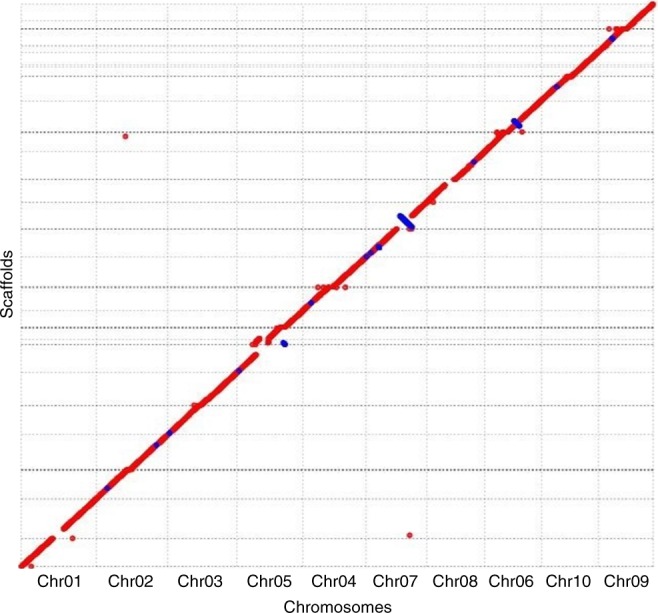
MUMmerplot comparison of *Tx430* hybrid scaffolds with the reference *BTx623* assembly. Hybrid scaffolds (*Y*-axis) were aligned to all 10 *BTx623* chromosomes (*X*-axis) using NUCmer and alignments were subsequently filtered for 1-on-1 alignments and rearrangements with a 20 Kbps length cutoff. Chromosome order on the *X*-axis is related to the alignment of *Tx430* scaffolds mapping to more than one chromosome. Chromosomal inversions and breakage in scaffold orientation in relation to the chromosomal sequence on the *X*-axis are shown as blue lines

To assess the completeness of the hybrid assembly, MUMmerplots were generated, following NUCmer v3.1 alignments of the 29 hybrid scaffolds described above to the v3.0.1 genome assembly, followed by filtering for 1-on-1 alignments and rearrangements with a 20 Kbps length cutoff. All 10 chromosomes from the public assembly were put in one unique plot (Fig. 3). Results suggested that the hybrid assembly overall exhibits a high degree of completeness in relation to the public genome assembly.

### Evaluation of the *Tx430* chromosome-scale hybrid assembly

The *Tx430* hybrid assembly scaffolds were first corrected via alignment to a *Tx430* 10X Genomics dataset (where the linked-read structure of the library was expected to preserve contiguity information between individual Illumina short reads) using the Long Ranger ALIGN pipeline, then polished with two rounds of RACON^28^. Scaffold polishing primarily corrected small indels, or mismatches with small indels nearby, that were missed by Pilon.

Several methods were used to determine the sequence accuracy of the hybrid scaffolds. First, regions identical by sequence between *Tx430* and *BTx623* (used to characterize Identity-By-Descent (IBD) regions between the two genotypes) were identified by mapping *Tx430* Illumina whole-genome shotgun reads to the v3.0.1 *S. bicolor* public assembly and calling SNPs between the two genotypes. Regions from the v3.0.1 assembly over 10 Kbps in length and located between SNPs were selected and aligned back to the *Tx430* combined assembly using minimap2. The alignments resulted in 10,468 *Tx430* regions totaling 127,154,880 bps. The mapping of *Tx430* Illumina whole genome reads to those 10,468 regions indicated the presence of 3833 remaining single-base mismatches, suggesting a sequence accuracy rate, excluding indels, of 99.997%. In a separate analysis, sequence accuracy was determined for the euchromatic regions only. The *Tx430* hybrid assembly was first masked using RepeatMasker^27^, and *Tx430* Illumina whole genome shotgun reads were aligned using Bowtie 2 against the 158,617 unmasked regions, totaling 127,535,027 bps. Only 2959 putative single-base mismatches were found, suggesting a sequence accuracy rate of 99.997%, again excluding indels. Finally, the *Tx430* 10X Genomics reads were assembled de novo with the Supernova assembler into 49,789 contigs, totaling ~613 Mbps. The resulting contigs were aligned to the hybrid assembly using NUCmer v3.1. Only contigs mapping to uniquely aligned clusters of 10Kbps or more (with a maximum of 10 bps gap between two adjacent matches within a cluster) were considered. Out of the 49,789 contigs produced by Supernova, 2225 of them, corresponding to ~322 Mbps, aligned. Sequence variations were detected by parsing the NUCmer alignments with dnadiff^29^. The number of high-quality mismatches (SNPs flanked by 20 bps perfect matches on both sides) decreased from 48,318 to 32,551, while the number of high-quality small indels decreased from 22,065 to 8188. Taken together, the final hybrid assembly exhibited an overall average sequence accuracy of 99.85%.

**Fig. 4:**
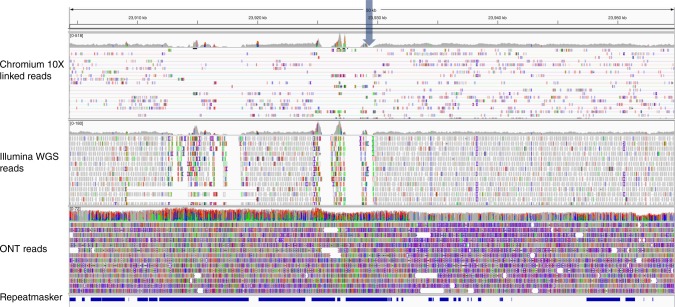
Close-up view of one inversion breakpoint area on Chromosome 6. A 50 Kbps region is shown, where Tx430 Chromium 10X linked reads, *Tx430* Illumina whole genome shotgun (WGS) reads, individual *Tx430* ONT reads aligning to the region and RepeatMasker screening output are shown, from top to bottom. The approximate location of the breakpoint area is marked by an arrow

The structural integrity of the large inversions found on chromosomes 6, 7, and 9 (Fig. 2) were assessed by aligning Illumina whole-genome shotgun reads (with BWA -MEM), 10X Genomics linked reads (with the Long Ranger ALIGN pipeline) and individual ONT reads (with BWA -MEM -x ont2d parameters) to the *Tx430* hybrid scaffolds. Alignments showed the presence of multiple ONT and 10X Genomics reads spanning the inversion breakpoints on chromosomes 6, 7, and 9 (Fig. 4 and Supplementary Figure 7a-e), confirming their structural integrity. RepeatMasker analysis showed that all breakpoint regions were in repeat-rich areas. Interestingly, a separate ~1 Mbp region present on chromosome 6 of the v1.0 *BTx623* genome assembly, subsequently corrected and re-assigned to chromosome 7^19,30^ was determined to be made of 72% repetitive DNA sequences.

Clusters of centromeric CEN38 motifs were detected on 19 hybrid scaffolds using BLAST, covering centromeric regions for all 10 chromosomes (Supplementary Figure 8a). Six of the chromosomes exhibited long clusters of CEN38 motifs while the remaining four had shorter clusters. Interestingly, many unmapped individual ONT contigs also contained long stretches of CEN38 motifs. Using similar BLAST search parameters, sub-telomeric STA1 and STA2 repeats^19^ were detected on hybrid scaffolds mapping to both ends of chromosomes 4 and 9. Arrays of STA1 and STA2 repeats also were found on scaffolds mapping to one end of chromosomes 2, 5, 7, 8, and 10. Finally, arrays of the (CCCTAAA)_n_ telomeric repeat were found in 17 chromosomal ends, slightly more than the 15 ends reported for the public v3.0.1 assembly^18,19^ (Supplementary Figure 8b).

As an initial assessment for overall genic completeness and accuracy, the 34,211 gene models from the public v3.0.1 release^19^ (corresponding to a mixture of bona fide protein-coding genes and a small number of ab initio predictions based on EST support from other crop species) were mapped using Bowtie 2 to the hybrid assembly. A total of 32,223 gene models, or 94.2%, were found to be entirely mapped to at least one scaffold, with a mean sequence identity of 98.75%. An additional 1857 transcripts (5.4%) were mapped partially to at least one scaffold, while only 132 transcripts were absent from the assembly.

Finally, the lengths of masked regions in the *Tx430* hybrid assembly were computed against their frequencies after searching for tandem repeats and masking the assembly with RepeatMasker. The majority of masked repetitive sequences were 2500 bps or less (Supplementary Figure 9). The longest masked sequence was 100,787 bps, confirming that assembled ONT reads can span large repetitive regions in plant genomes.

### Generation of a *BTx623* Bionano DLS contig map

**Fig. 5:**
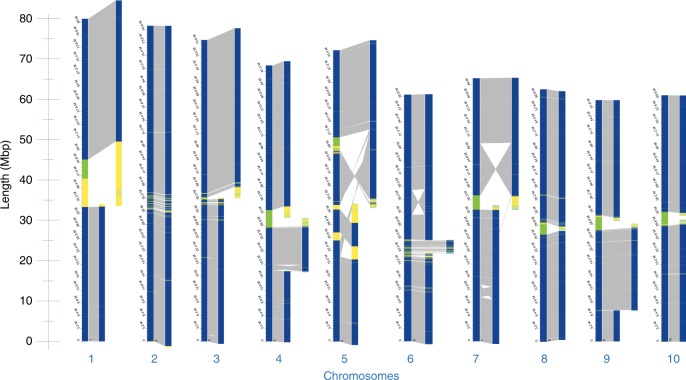
Alignment of *BTx623* maps against in-silico maps of the *BTx623* reference assembly. Large inversions were detected on chromosomes 6 and 7 after aligning the *BTx623* DLS map to in silico maps derived from the v3.0.1 public *BTx623* assembly. A third inversion was detected on chromosome 5 while a separate inversion originally detected on chromosome 9 (Fig. 2) was absent in *BTx623*. Chromosomes are listed in numerical order. Regions in green are stretches of random nucleotides in the reference assembly. Regions in yellow exhibit breaks in collinearity between the two maps

A Bionano DLS optical map was generated for *BTx623* to determine the origin of the structural discrepancies between the *Tx430* DLS optical map and the in silico map derived from the *BTx623* v3.0.1 genome assembly. The de novo assembly yielded 44 maps, with a resulting map N_50_ of 34.61 Mbps (Supplementary Table 5). Surprisingly, comparisons between the *BTx623* DLS optical map and the in-silico map created from the *BTx623* v3.0.1 genome assembly (Fig. 5) confirmed the two major inversions found on chromosomes 6 and 7 in *Tx430*. A third large inversion, not initially detected on the *Tx430* DLS map due to its fragmented nature in that region, was also detected on *BTx623* chromosome 5. On the other hand, a fourth 608 Kbps inversion initially detected on chromosome 9 in *Tx430* was not detected in *BTx623*, suggesting the existence of a chromosomal inversion between the two genotypes in that region. Further studies that are beyond the scope of this paper may be warranted to determine the origin of the structural discrepancies between the v3.0.1 genome assembly and the *BTx623* DLS optical map. However, as noted earlier, another inversion in the original *S. bicolor* v1.0 genome assembly had been previously reported^19,30^. In addition, the inversion breakpoints reported here showed extensive coverage by spanning individual Bionano individual molecules (Supplementary Figure 10).

### Annotation of the *Tx430* hybrid assembly

Genes, tRNAs, and repeats were predicted as part of the *Tx430* genome annotation procedure. Gene calling was performed using a hybrid approach with de novo gene predictors and evidence-based methods, generating a total of 39,510 genes coding for 53,240 transcripts, of which 52,353 were protein-coding and the rest were tRNA genes. Table 3 shows a comparison of annotation between v3.0.1 (*BTx623*) and *Tx430*. More protein-coding genes and more transcripts per gene were predicted in *Tx430* when compared to *BTx623*. However, the *BTx623* annotation predicted slightly longer genes with a higher median exon number. A sizable number of UTRs not established in the *BTx623* annotation were also predicted in *Tx430*. A BUSCO comparison between *BTx623* and *Tx430* (Supplementary Figure 11) showed that more than 98% of the BUSCO’s were accounted for in both annotations (including 97.8% and 99.1% complete in either single-copy or duplicated form in *Tx430* and *BTx623*, respectively), even though *Tx430* had a slightly higher number of fragmented and missing BUSCO’s than *BTx623* (a more thorough annotation utilizing experimental data from *Tx430* might clear the case of the missing and fragmented BUSCO’s, but is presently beyond the scope of the study).

**Table 3:** Annotation comparison metrics

	*BTx623*	*Tx430*
Number of genes	32,945	39,510
Number of mRNAs	39,712	53,240
Number of protein-coding genes	28,244	38,623
Number of proteins	39,248	52,353
Number of 5′ UTRs	NA	41,950
Number of 3′ UTRs	NA	40,682
Median gene length	2923	2031
Median cDNA length	3585	2827
Median CDS length	1179	1086
Median intron length	156	148
Median exon length	163	162
Median exon number	4	3
Median protein length (a.a.)	393	362
Overlapping genes	2577	2635
Contained genes	570	433
Number of non-coding genes	4709	887
Number of unique proteins	34,076	47,133
Number of unique protein domains	4862	5978
Percentage of genome covered by genes	18.5	18.3

Lengths are given in bps, unless otherwise noted. *a.a.* amino acids, *NA* not available

A reciprocal best BLAST hit analysis between *Tx430* and *BTx623* proteins reveals that ~95% of the *BTx623* proteins are homologous to ~78% of *TX430* proteins. Of the remaining 11,412 proteins, 3301 were of bacterial origin and were removed from further analyses. The unmapped ONT contigs carrying those genes and determined to be of plant pathogens as described earlier were also removed from the final *Tx430* assembly scaffold and contig set. Of the remaining 8111 proteins, 6589 did not have a known Pfam domain associated with them. They were largely uncharacterized (2073), or putative novel proteins (3591) without any functional description. The remaining 1522 proteins had one of 893 unique domain architectures associated with them. A total of 428 unique domain architectures in 718 proteins were found to have a GO classification. Supplementary Figure 12 shows the GO enrichment in these 718 proteins w.r.t. a biological process, Supplementary Figure 13 shows the same for molecular function, and Supplementary Figure 14 shows the GO enrichment for cellular component. Gene ontology terms GO:0003676 (nucleic acid binding), GO:0008270 (zinc ion binding) and GO:0006355 (regulation of transcription) accounted for more than 50% of these 718 proteins, suggesting that several DNA-binding transcription regulators, metal-ion transporters, vesicular associated proteolytic enzymes were unique to *Tx430* and could be attributed to PAV variation among lines.

We compared the NB-ARC and LRR domain-containing proteins between *Tx430* and *BTx623* as an additional indicator of the quality of annotation. We found 241 and 194 NBS-LRR domain proteins in *Tx430* and *BTx623*, respectively. Reciprocal BLAST analysis of the two protein datasets indicated that 97% of the proteins were global homologs of each other, indicating that the annotation strategy recovered most of the Resistance Gene Analogs (RGA) type genes in *BTx623*. Seven genes in *Tx430* and five genes in *BTx623* were unique NBS-LRR genes in these lines.

## Discussion

This study demonstrates that the Oxford Nanopore sequencing technology, combined with the DLS optical mapping technology recently developed by Bionano Genomics, can generate chromosome-scale assemblies of large and repeat-rich plant genomes. The data presented here demonstrate that ONT sequences alone can rapidly generate highly accurate and informative sorghum assemblies and that ultra-long-range genomics technologies such as Bionano Genomics optical maps can lead to chromosome-scale assemblies of a complex plant genome. DLS optical maps were used in the present study. Because labeling occurs directly without introducing nicks in the DNA strands, the average length and overall N_50_ of DLS optical maps tend to be significantly longer than the ones obtained using nicking enzymes, which further facilitated correcting, orienting, and scaffolding of the ONT contigs. Alignments of the DLS optical maps generated in this study to the *S. bicolor* reference genome showed that their sizes are mostly limited by the size of chromosomes. With sufficient sequencing coverage, ultra-long ONT reads (including reads over 1 Mbp in length) may represent a useful complement to DLS optical maps. Similar N_50_ values as the one obtained here have been predicted in human when using ultra-long ONT sequencing reads at ~30× sequencing coverage^6^.

The *Tx430* annotation exhibits good congruency with the public v3.0.1 *BTx623* annotation. It must be noted that the annotation of the *BTx623* genome assembly was facilitated by combining short-read cDNA (RNA-Seq) datasets from multiple tissues or development stages with sorghum ESTs and homology-based prediction software^19^. Therefore, the increased number and shorter median length of *Tx430* protein-coding genes, whose structure could be further refined through alignments and comparisons with deep transcriptome assemblies derived from *Tx430* RNA-Seq datasets, might be related to the presence of genes absent from the *BTx623* genome, but also to the presence of short indels and mismatches remaining in the final *Tx430* hybrid assembly. Those discrepancies in turn, might create false stop codons or frameshift mutations, and the emergence of falsely truncated genes. Nonetheless, the *Tx430* annotation metrics and comparisons to the BUSCO’s and public *BTx623* gene datasets suggest that a majority of the *BTx623* protein-coding genes are present in the *Tx430* assembly.

The *BTx623* DLS optical map contigs, combined with the analysis of *Tx430* ONT and 10X Genomics linked-read data in regions of interest, suggest that large pericentromeric inversions, such as the ones found on chromosomes 6 and 7 correspond to assembly errors in the v3.0.1 reference genome. However, additional analysis will be required for confirmation. Interestingly, the ~1 Mbp region present on chromosome 6 of the v1.0 *BTx623* genome assembly and subsequently re-assigned to chromosome 7 of the v3.0.1 *BTx623* genome assembly^19,30^ is in a region adjacent to an apparent intra-chromosomal translocation event observed between the *Tx430* and *BTx623* DLS maps generated for this study and the public *BTx623* v3.0.1 genome assembly. As the ~1 Mbp region re-assignment was based on high-density genetic marker information^30^, it is unlikely that the structural discrepancies observed in this region are due to assembly errors. Therefore, the use of a *BTx623* seed source different from the one used for the public *BTx623* genome assembly project could explain at least some of the variations observed with the public v3.0.1 genome assembly. Despite those observations, the current state of the assembly confirms that working-draft assemblies can be achieved rapidly for large and complex plant genomes using long-read nanopore sequencing technologies, and chromosome-scale scaffolding of those working draft assemblies can be achieved through DLS optical mapping technologies. These results have multiple implications. First, by greatly enhancing the contiguity of ONT assemblies, DLS optical maps are expected to greatly facilitate comparative genomics studies at the chromosomal level, and therefore accelerate pan-genomic analysis between multiple genotypes in a species of interest^31^. Pericentromeric inversions, such as the ones detected on chromosome 9, could impact marker detection and recombination in a breeding program involving parental lines distinguished structurally by such inversions. Contig sizes attained in this study also could facilitate the direct comparison of regions involved in specific traits of interest, such as disease-resistance gene clusters. Second, sequences in this study were generated using the USB-sized MinION sequencer. Its overall portability means that informative whole-genome assemblies can now be attained in multiple settings and locations that, up until recently, did not have the means and resources to generate such assemblies. The de novo assembly of a complex plant genome also could be greatly accelerated by the adoption of larger ONT sequencing platforms, including the PromethION where up to 48 flow cells, each generating more than 100 Gbps of data, can be run simultaneously. This could have a remarkable impact on how genomes are being determined and defined in the world of agricultural biotechnology, by allowing the rapid characterization, for example, of mutagenized lines or of transgene insertions, or by facilitating the design of multiple gene editing targets and allowing the direct prediction of potential off-target mutations in a large number of transformed plants.

## Methods

### Plant material and genomic DNA extraction

Whole leaves were sampled from *S. bicolor* accession *Tx430* grown in the greenhouse for approximately 2 months. Samples were immediately collected in dry ice and stored at −80 °C until further handling. 25–30 g of frozen tissue was ground in liquid nitrogen prior to extraction of High Molecular Weight (HMW) genomic DNA from sorghum *Tx430* adult leaf tissue using a protocol which was originally designed for the extraction of DNA for BAC library construction^20^. For Bionano mapping, fresh tissue was collected from 3-week-old seedlings. HMW genomic DNA was prepared according to a protocol developed specifically by Bionano Genomics. Briefly, 2 g young leaf tissue was collected from live plants and immediately treated with a formaldehyde solution. After fixation, tissue was cut into approximately 2 × 2 mm squares and homogenized using a stator. Nuclei were isolated by gradient centrifugation and embedded into agarose plugs. After overnight proteinase K digestion in the presence of Lysis Buffer (Bionano Genomics) and 1 h treatment with RNase A, plugs were washed three times in 1× Wash Buffer (Bionano Genomics), then five times in 1× TE buffer. After the final wash, DNA was recovered by melting the plugs, digesting agarose with 2 µl of 0.5 U/µl Agarase, and dialyzing for 45 min in 1× TE buffer.

### Oxford Nanopore MinION library construction and sequencing

For short read MinION runs, 2–3 µg HMW DNA was sheared to ~8 Kbps with a g-Tube (Covaris) prior to library construction. For long read MinION runs, up to 100 µl HMW DNA at a concentration not exceeding 50 ng/µl was size-selected to 20 Kbps and above using a PippinHT system (Sage Science). The DNA recovered from the instrument was quantified and used directly for library construction.

Short read libraries and all but three long read libraries were built using the Ligation Sequencing Kit 1D (SQK-LSK108) from Oxford Nanopore. Briefly, DNA repair first was performed by incubating 2–4 µg of genomic DNA using a FFPE Repair Mix enzymes (New England Biolabs) in 1× FFPE Repair Buffer for 15 min at 20 °C, followed by cleanup with 0.5× AMPure XP beads and resuspension of the DNA in 45 µl nuclease-free water. (For DNA samples with an initial DNA concentration lower than 45 ng/µl, multiple DNA repair reaction, each containing 45 µl of DNA, were performed and pooled together after repair and before bead cleanup.) End repair and dA-tailing were then performed simultaneously by mixing repaired DNA with 3 µl Ultra II End-prep enzyme mix (New England Biolabs) in 1× Ultra II End-prep reaction buffer, and incubating 20 min at 20 °C, followed by 20 min at 65 °C. After cleanup with 0.5× AMPure XP beads, DNA was ligated with 1D Adapter Mix (Oxford Nanopore Technologies) in 1× Blunt/TA Ligation Mastermix (New England Biolabs) and incubated for 10 min at 20 °C. Cleanup was performed by resuspending ligated DNA with 0.5× AMPure XP beads and washing the beads twice with 140 µl Adapter Bead Binding buffer (Oxford Nanopore Technologies). After washing, DNA was dried down and resuspended in 12 µl of Elution Buffer (Oxford Nanopore Technologies) before being combined with 35 µl RBF buffer (Oxford Nanopore Technologies) and 28 µl nuclease-free water and loaded on a MinION SpotON flow cell (FLO-MIN106). Sequencing was performed for 48 h with a MinION Mk1B sequencer. The resulting FAST5 files were base called with v2.0.1 of the Albacore base caller. The remaining three long read libraries were prepared with the ONT Rapid Sequencing Kit 1D (SQK-RAD002). Briefly, 16 µl of HMW genomic DNA (corresponding to 2–4 µg of DNA, less than the recommended 16 µg of DNA) was mixed with 5 µl FRN fragmentation mix (Oxford Nanopore Technologies) and incubated at 30 °C for 1 min, followed by 75 °C for 1 min. After incubation, 1 µl Rapid 1D Adapter mix (Oxford Nanopore Technologies) and 1 µl Blunt/TA Ligation Mastermix (New England Biolabs) were added and the library as incubated at 20 °C for 30 min. Loading on a MinION flow cell (FLO-MIN106) was performed after mixing the library with 25.5 µl RBF buffer (Oxford Nanopore Technologies) and 27.5 µl nuclease-free water.

### Illumina sequencing

Illumina whole-genome shotgun sequencing was performed using the same HMW DNA as the one used for MinION sequencing. Briefly, after randomly shearing ~1 µg of DNA to ~600–700 bps with a Covaris S2 Focused Ultrasonicator (Covaris), a whole-genome shotgun paired-end Illumina library was prepared using a KAPA Hyper Prep Kit (KAPA Biosystems), following the manufacturer’s recommendations, and sequenced on an Illumina HiSeq2500 to approximately 150× sequencing coverage equivalent of the sorghum genome. The resulting reads were trimmed and quality-filtered before polishing.

A 10X Genomics Chromium library was build using a separate HMW DNA aliquot, following the manufacturer’s instruction, and sequenced on an Illumina HiSeq2500 to approximately 60× sequencing coverage of the sorghum genome. The resulting reads were subsequently trimmed and quality-filtered. Sequencing reads were later aligned using the Long Ranger ALIGN pipeline (10X Genomics) and assembled using the Supernova de novo assembler (10X Genomics).

### Oxford Nanopore MinION genome assembly and analysis

Oxford Nanopore MinION runs were base called using ONT Albacore Sequencing Pipeline Software (version 2.0.1) on the FAST5 files. Passed reads (*Q* score ≥ 7) of length 2K+ bp from all MinION runs were fed into Canu (version 1.6) for read correction, followed by de novo assembly using SMARTdenovo (https://github.com/ruanjue/smartdenovo) on the corrected reads of length 5K+ bp. Canu correction was run by setting corMaxEvidenceErate to 0.15 and correctedErrorRate to 0.12, while SMARTdenovo was run by setting the engine of overlapper to compressed kmer overlapper and the generate consensus to true. The SMARTdenovo consensus sequences were then polished with 100× Illumina reads using Pilon (version 1.21) for two iterations. RACON was also applied to contig polishing but only on the scaffolded hybrid assembly. Tandem repeats in non-scaffolded ONT contigs were detected using the Tandem Repeats Finder (http://tandem.bu.edu/trf/trf409.linux64.download.html).

*Tx430* regions of similar haplotype between *Tx430* and *BTx623* (labeled as IBD or identity-by-descent) were determined by aligning Illumina WGS reads from *Tx430* to the public *BTx623* reference genome using Bowtie 2, calling SNPs on *BTx623* if the read coverage depth was greater than 2 and the major allele frequency was greater than 97%, identifying non-N regions without any SNPs, and filtering out regions with no read coverage depth. Sequences of IBD regions of length greater than 10 Kbp were then extracted from *BTx623* and mapped to *Tx430* assembly using minimap2. Alignments on *Tx430* of coverage greater than 90% were identified as the corresponding IBD regions on *TX430*. In total, there were about 6600 IBD regions of 10Kbp+ identified on *Tx430*, with a total size of 127 Mbps.

The accuracy and completeness of polished contigs were evaluated by two methods. Full-length contigs were mapped to the public reference v3.0.1 using NUCmer 3.1 (MUMmer v3.9.4alpha) and the completeness comparison was performed by MUMmerplot 3.5 (MUMmer 3.23 package) on the NUCmer results after filtering to 1-on-1 alignments and allowing rearrangements with a 20 Kbps length cutoff. Contigs were also chunked into 10 Kbp fragments and mapped to the public reference using minimap2 (version 2.10) by allowing at most 3 secondary alignments. Average identity was calculated for the best hit of 50+% coverage for every chunk. To further evaluate the accuracy of polished contigs, Illumina reads were mapped to the contigs using Bowtie 2 and the average identity was calculated using uniquely mapped reads. Illumina reads were also mapped to the public reference v3.0.1 using Bowtie 2 for accuracy comparison. Lastly, the gene model set from the public reference v3.0.1 was mapped to the polished contigs using Bowtie 2 to evaluate the completeness of genic sequences coverage.

### DLS optical maps construction and hybrid assembly

Labeling and staining of the DNA were performed according to a protocol developed by Bionano Genomics. Labeling was performed by incubating 750 ng genomic DNA with 1× DLE-1 Enzyme (Bionano Genomics) for 2 h at 37 °C, followed by 20 min at 70 °C, in the presence of 1× DL-Green (Bionano Genomics) and 1× DLE-1 Buffer (Bionano Genomics). Following proteinase K digestion and DL-Green cleanup, the DNA was pre-stained by mixing the labeled DNA with 1× Flow Buffer (Bionano Genomics), in the presence of 1× DTT, and incubating overnight at 4 °C. Staining was performed by adding 3.2 µl of a DNA Stain solution (Bionano Genomics) for every 300 ng of pre-stained DNA and incubating at room temperature for at least 2 h before loading onto the Bionano Chip. Loading of the chip and running of the Bionano Genomics Saphyr System were all performed according to the Saphyr System User Guide (https://bionanogenomics.com/support-page/saphyr-system/). Data processing, construction of the DLS contigs and of the hybrid map assembly were all performed using the Bionano Genomics Access software suite.

### Annotation of the *Tx430* hybrid assembly

Genome annotation was carried out by first masking the repeats using RepeatMasker (http://www.repeatmasker.org/) and a curated sorghum specific repeats file from Repbase (https://www.girinst.org/repbase/). The repeat-masked genome was used as input to two categories of gene predictors. De novo gene prediction programs Fgenesh (licensed Ver 7.2.2), Augustus (Ver. 2.7 http://bioinf.uni-greifswald.de/augustus/) and SNAP (Ver. 2006-07-28 https://github.com/KorfLab/SNAP) were run under default parameters and the training sets used were monocots, maize, and rice respectively. EST, cDNA, long read evidence-based gene structure modelers GMAP (Ver. 03-25-2018 http://research-pub.gene.com/gmap/), and PASA (Ver. 2.2.2 https://github.com/PASApipeline/PASApipeline/wiki) were run with the *–max-intron-size* parameter adjusted to 20,000. Protein evidence-based gene structure modeler SPLAN (Ver. 2.1.3 http://www.genome.ist.i.kyoto-u.ac.jp/~aln_user/spaln/) was run under default conditions. Long read sequences of *BTx623* line of sorghum from NCBI, along with other sorghum EST’s and cDNA were used as the evidence set to PASA. Other non-sorghum Poales EST, cDNA sequences from NCBI, and monocot transcripts from phytozome were used as additional closely related species evidence for gene prediction with GMAP (*parameters –min-identity 80 –min-coverage 95*). Uniref100 plant protein sequences were used as evidence dataset for gene structure prediction using SPLAN. All gene annotation files were run through EvidenceModeler and the output used to polish the gene boundaries in PASA. The final PASA annotation file was combined with tRNA predictions file from tRNA-ScanSE to obtain the final structural annotation file, along with fasta sequences of protein, CDS, cDNA and gene.

Pfam domain architectures for each protein were found using hmmscan against the Pfam database. A psiblast search against the entire Uniport database yielded the functional description for each protein. General protein properties like molecular weight, amino acid composition, and average hydropathy index were calculated using Biopython modules. Signal-P and TM-HMM programs were used to predict N-terminal localization signals and transmembrane domains of the proteins respectively. The function predictions were then replicated in the final annotation gff3 file, as well as in the corresponding gene, cDNA and CDS sequence files. The annotation was visualized in JBrowse. Statistical analyses and comparison were performed using the Genome Annotation Generator package (https://genomeannotation.github.io/GAG/), and GenomeTools (http://genometools.org/tools.html). BUSCO analysis for annotation quality assessment was performed using the BUSCO version 3 software (https://busco.ezlab.org/) and GO enrichment analysis was performed using REVIGO (http://revigo.irb.hr/).

### Reporting summary

Further information on research design is available in the Nature Research Reporting Summary linked to this article.

## Electronic supplementary material

Supplementary Information

Reporting Summary

## Data Availability

All data that support the findings of this study, including raw sequencing data, raw Bionano Genomics molecules and assembled maps, and the final polished assembly, have been deposited in the National Center for Biotechnology Information database under the BioProject accession number PRJNA472170. The Whole Genome Shotgun project has been deposited at DDBJ/ENA/GenBank under the accession QWKM00000000. The version described in this paper is version QWKM01000000. Raw sequencing data have been deposited under SRA accession number SRP148505. All other relevant data are available from the corresponding authors on request. A reporting summary for this Article is available as a Supplementary Information file.
