# Supplementary material for: A chromosome-scale assembly of the sorghum genome using nanopore sequencing and optical mapping
Source: Nat Commun. 2018 Nov 19;9:4844. doi: 10.1038/s41467-018-07271-1 (PMC6242865; doi:10.1038/s41467-018-07271-1)
Supplement: Supplementary file 1 — Supplementary Information [file 41467_2018_7271_MOESM1_ESM.pdf]

# A chromosome-scale assembly of the sorghum genome using nanopore sequencing and optical mapping

Deschamps et al.

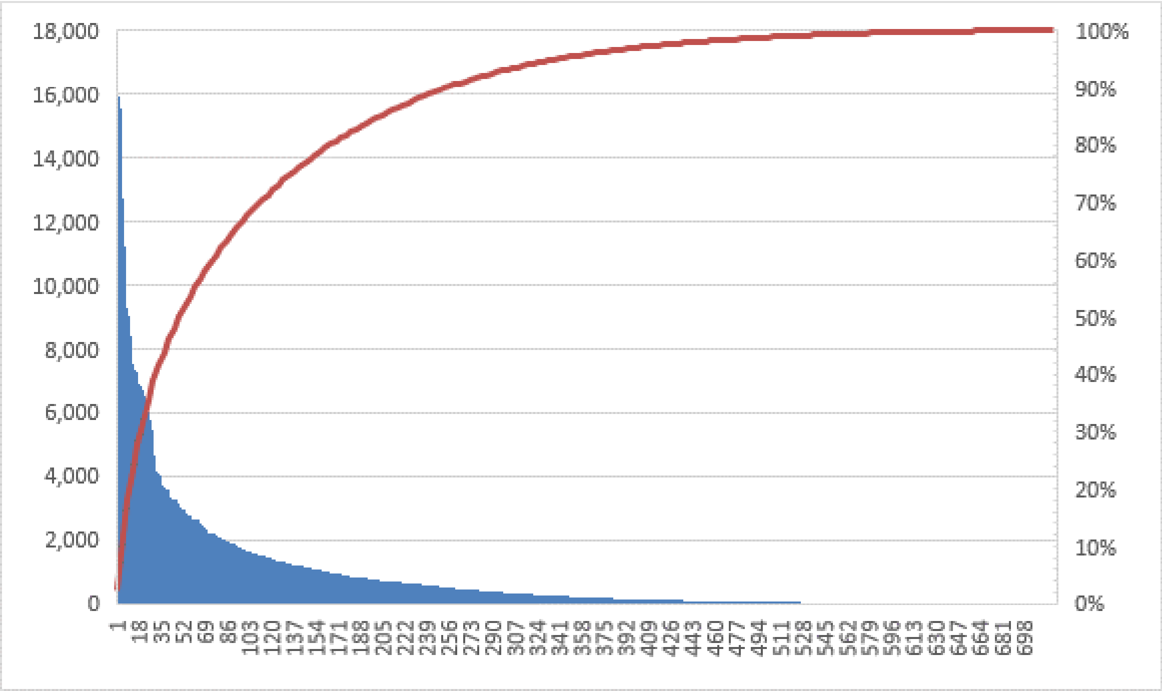


**Supplementary Figure 1: Length distribution of ONT contigs after two rounds of polishing with Pilon.** Contig size distribution is shown in blue, corresponding to the Y-axis on the left. % completion of the total assembly size is marked by a red line and corresponds to the Y-axis on the right.

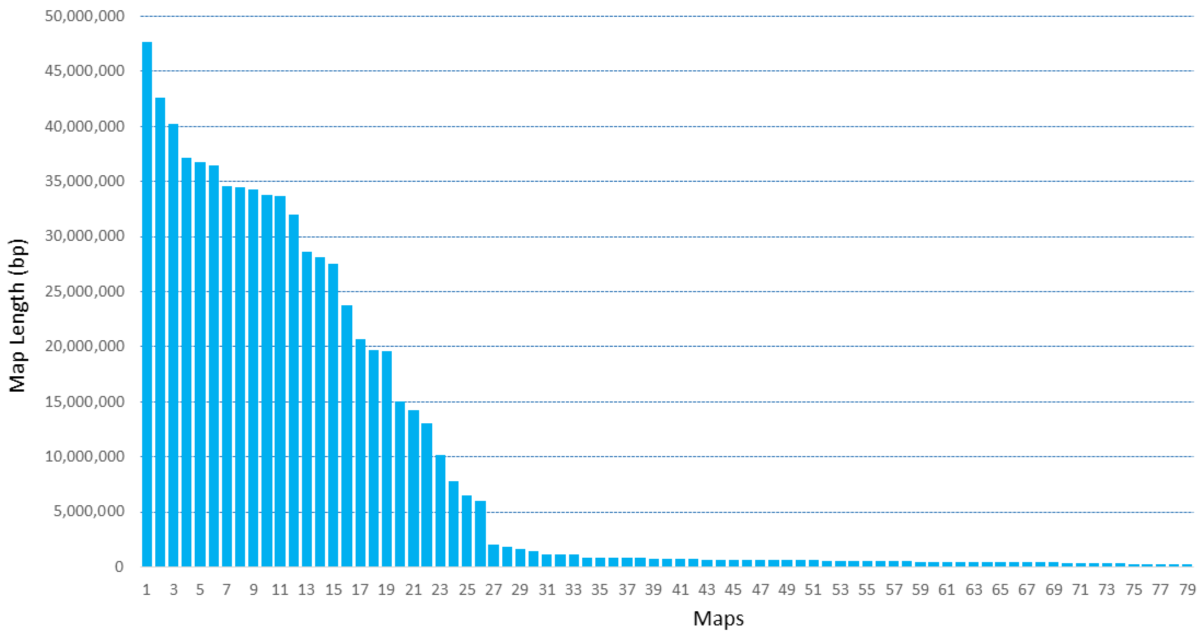


**Supplementary Figure 2: Summary of de novo assembly of single molecules into DLS optical maps.** A total of 79 maps were created, out of which 32 accounts for 99.5% of the expected assembly size. The assembly $N_{50}$ is 33.77Mbps while the largest map was 47.64Mbps in length.

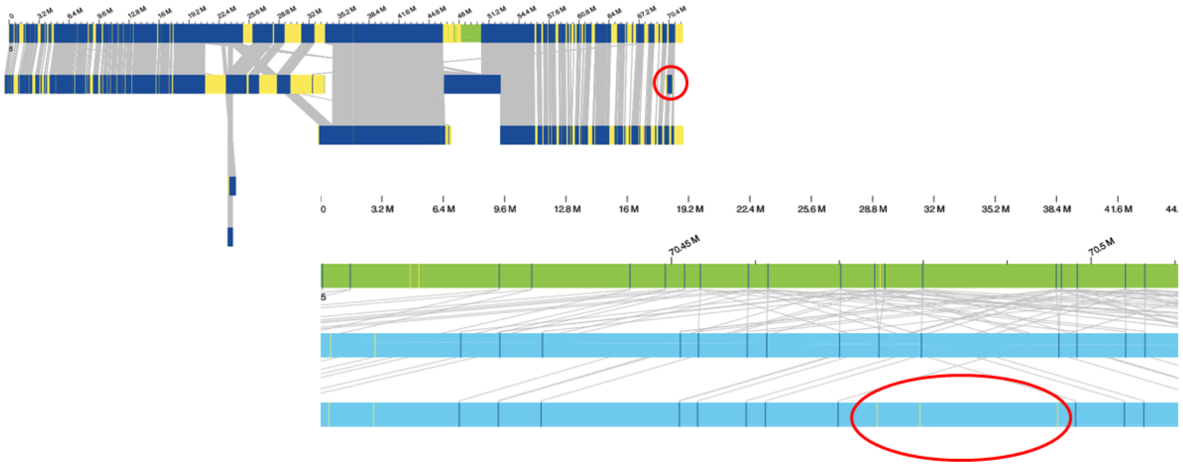

**Supplementary Figure 3: Tx430 chromosome 5 and close-up view of overlapping contig.** (Upper) Tx430 chromosome 5: v3.0.1 public assembly (top row); DLS optical maps (middle and bottom rows). The overlapping DLS contig is marked by a red circle. (Lower) Close-up view of the overlapping region (details): hybrid scaffold is shown in green. DLS maps are shown in blue. Circles indicate regions of potential heterozygosity on Tx430 chromosome 5 hybrid assembly, corresponding to unmapped DLE-1 motifs, shown in yellow on the DLS maps.

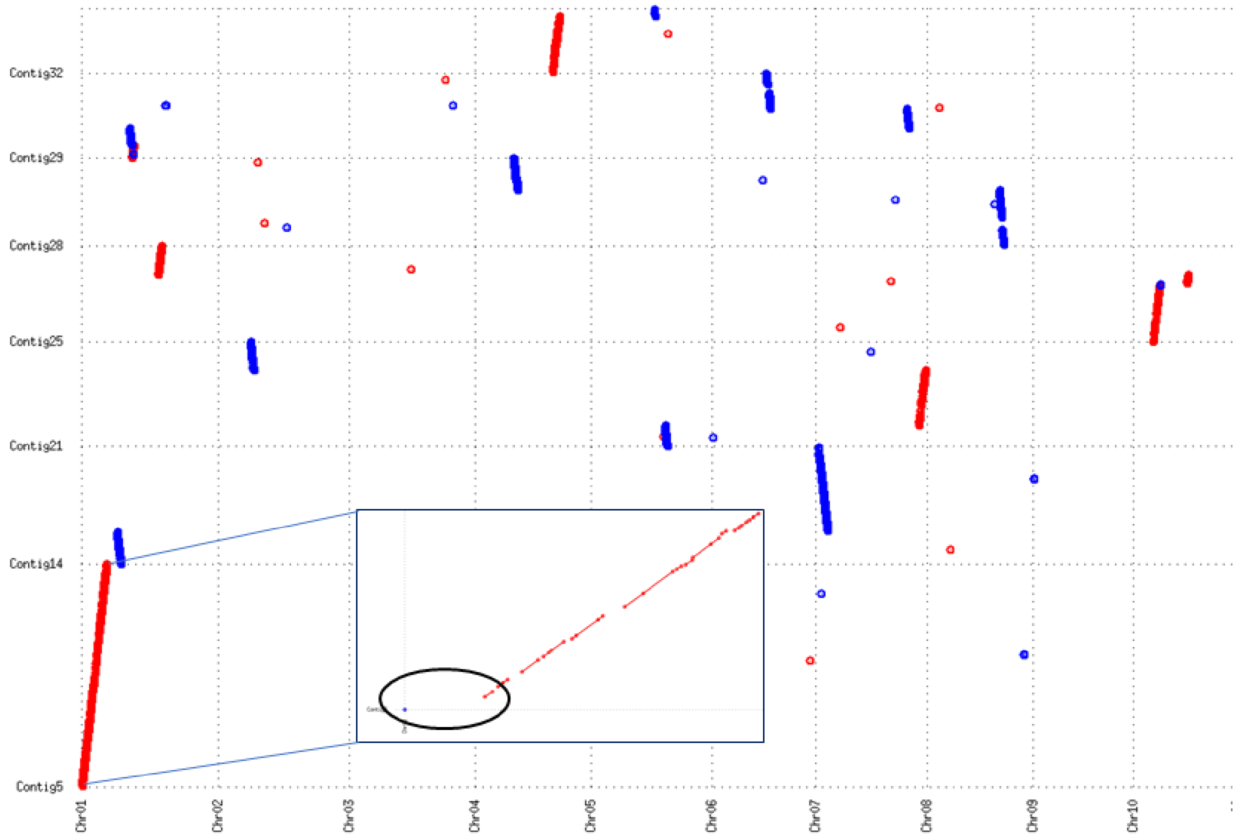


**Supplementary Figure 4: NUCmer v3.1 alignments of selected chimeric ONT contigs to the v3.0.1 reference assembly.** A subset of all ONT contigs were split into smaller contigs by the Bionano software during hybrid assembly generation. Locations of the splits and the subsequent location of the resulting smaller contigs were confirmed by way of alignment to the v3.0.1 assembly. (X-Axis) v3.0.1 chromosomes; (Y-Axis) a subset of corrected ONT contigs before correction and hybrid assembly generation. Contigs are listed by numbers as determined by the Bionano software. Sequence alignments are shown. The color represents the orientation of the alignment. A close-up version of the split that occurred on the ONT contig mapping to v3.0.1 Chromosome 1 (“Contig5”) is shown.

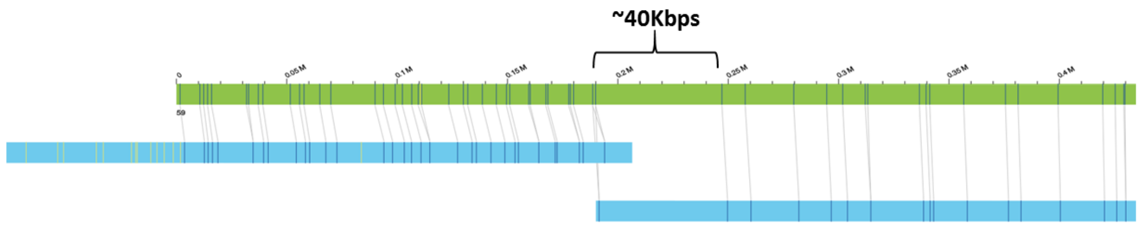

**Supplementary Figure 5: Chimeric assembly of Bionano contig maps (details).** The ends of two Bionano contig maps (in blue) are merged by the presence of overlapping ONT contig sequences at their respective ends (shown in green), forming a chimeric hybrid map. The chimeric map was corrected by manually deleting the ~40Kbps ONT contig sequence merging the ends of the two Bionano contig maps.

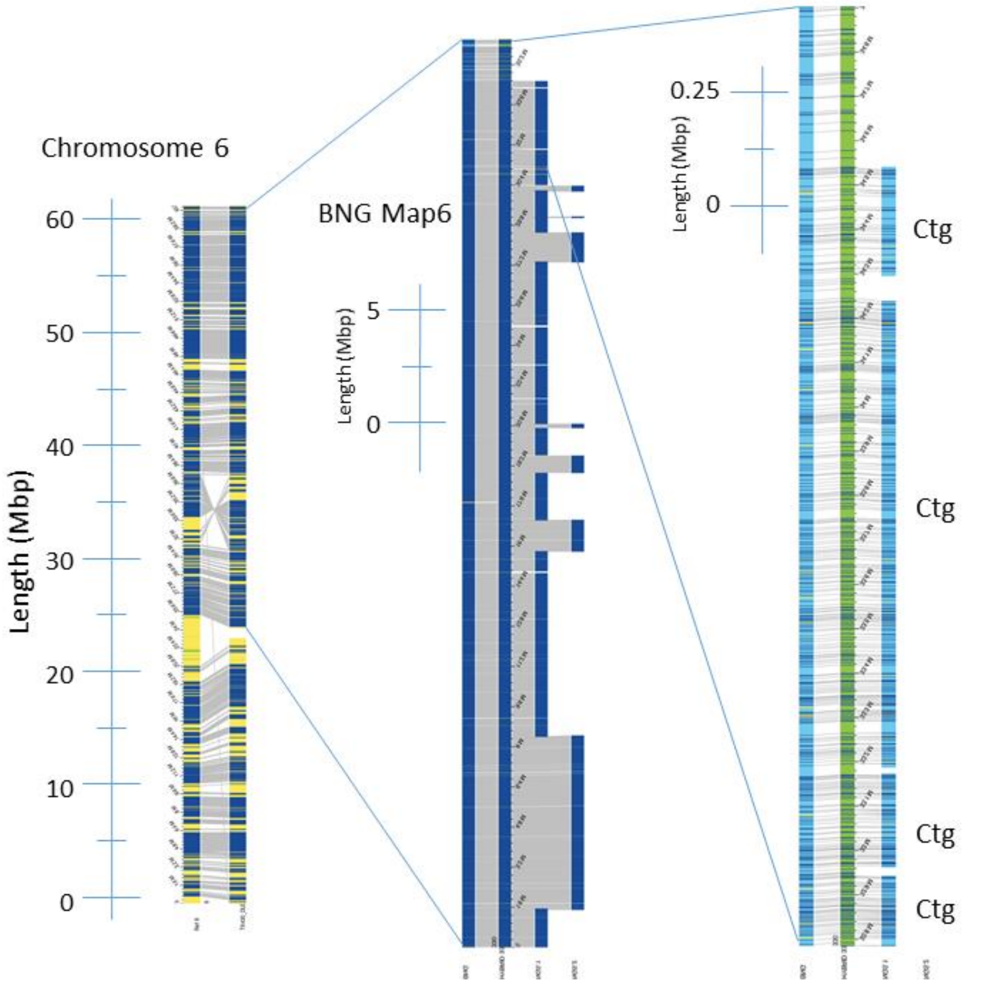


**Supplementary Figure 6: Detailed view of a region of Chromosome 6 and corresponding DLS maps.** Left: DLS maps aligning to *in-silico* maps of Chromosome 6 from public v3.0.1 assembly; Center: Close-up view of DLS map 6 and mapped ONT contigs; Right: Close-up views of hybrid maps (center – green) generated by merging DLS maps (left – blue) and in-silico maps of ONT contigs (“Ctg”, right – blue). Distances are shown, in Mbps.

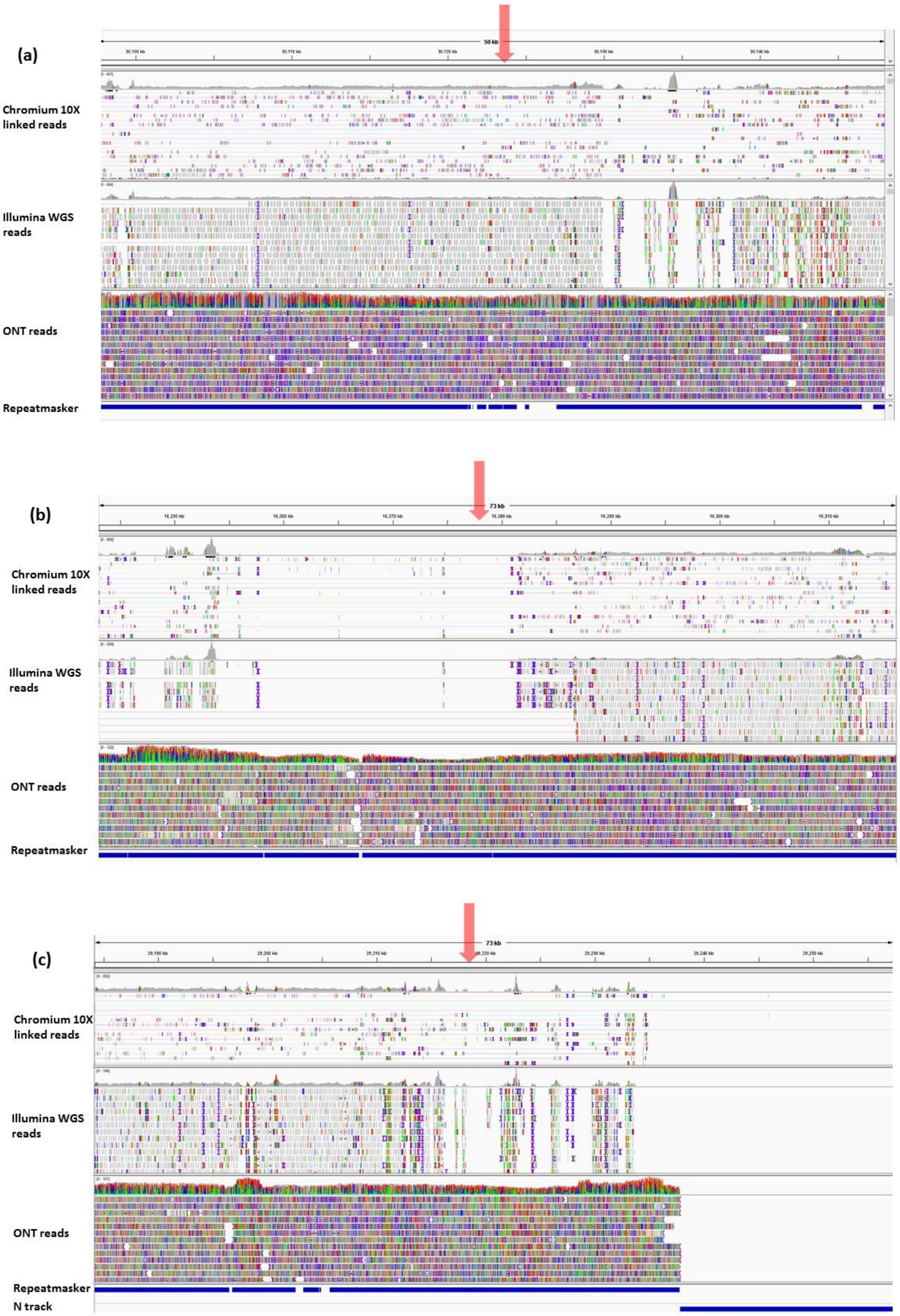

(a)
50 kb
Chromium 10X linked reads
Illumina WGS reads
ONT reads
Repeatmasker
(b)
73 kb
Chromium 10X linked reads
Illumina WGS reads
ONT reads
Repeatmasker
(c)
73 kb
Chromium 10X linked reads
Illumina WGS reads
ONT reads
Repeatmasker
N track

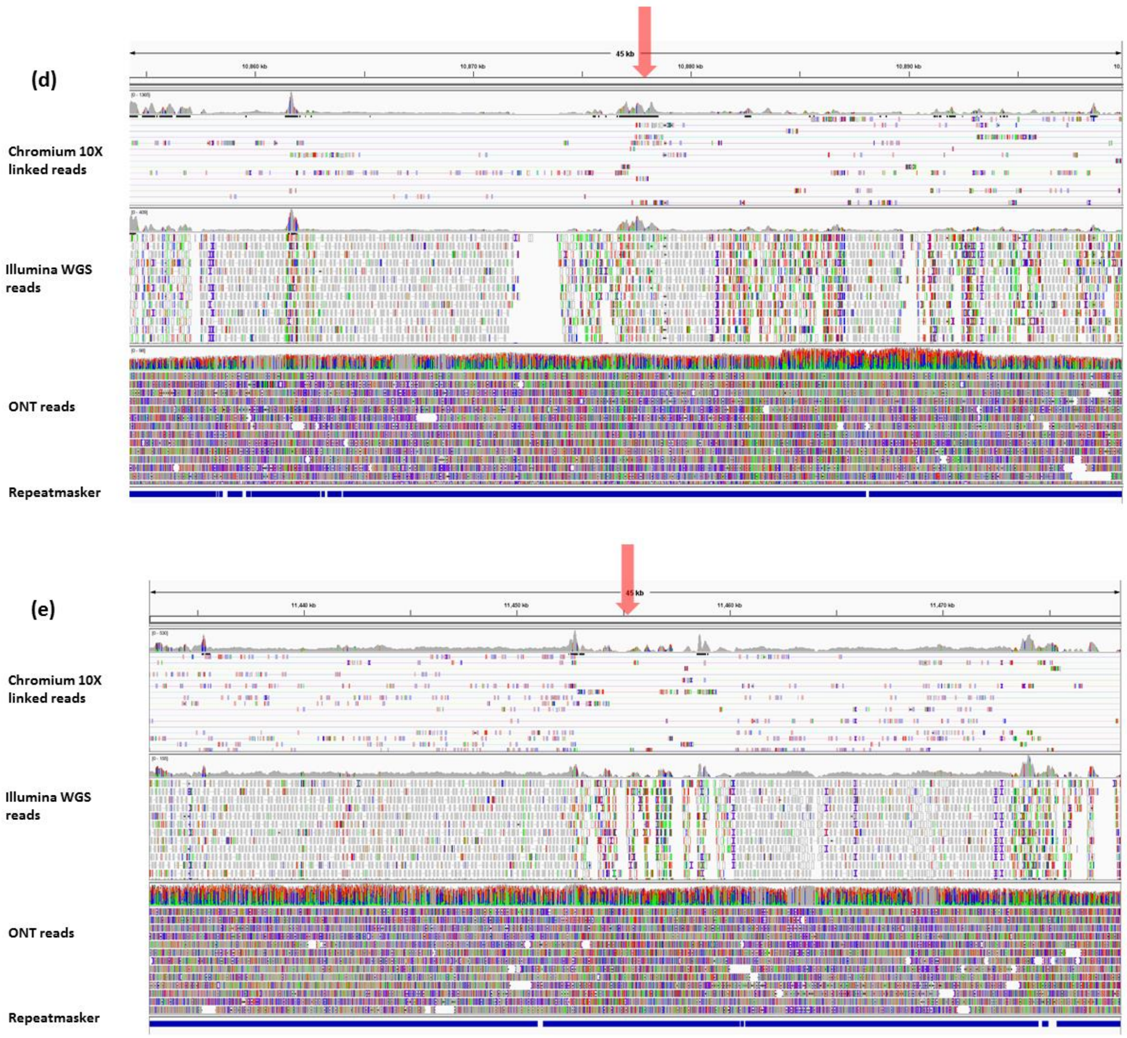


**Supplementary Figure 7: Close-up view of the inversion breakpoints located on Chromosome 6, 7 and 9.** TX430 Chromium 10X linked reads, Tx430 Illumina whole genome shotgun (WGS) reads, individual Tx430 ONT reads aligning to the breakpoint region, as well as RepeatMasker output and stretches of N's in the hybrid Tx430 scaffolds are shown, from top to bottom. The approximate locations of the inversion's end are marked by an arrow. (a) Chromosome 6; (b) and (c) Chromosome 7; (d) and (e) Chromosome 9.

(a)

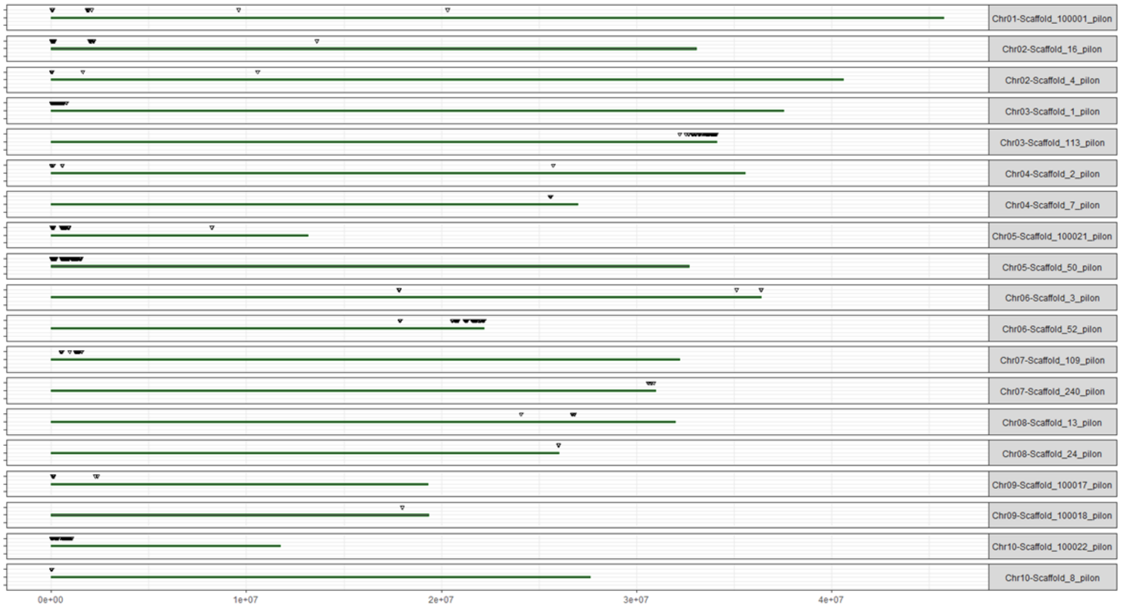


(b)

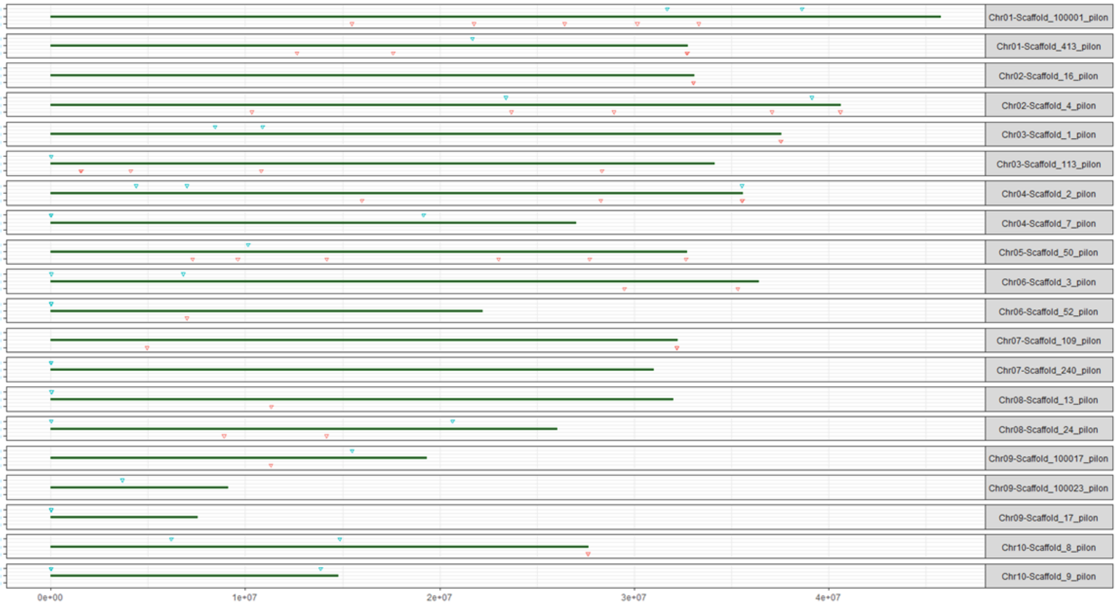


**Supplementary Figure 8: detection of centromeric CEN38 and telomeric (CCCTAAA)$_n$ motifs on hybrid scaffolds.** (a) Clusters of CEN38 motifs (at least 50 CEN38 hits per clusters; >80% coverage; >80% identity) and their locations are marked by a black triangle on each scaffold. (b) (CCCTAAA)$_n$ motifs (n=4; 28bps perfect match) are marked by a blue or red triangle on each scaffold, depending on their orientation. Telomeric motifs are found in regions mapping to one end of chromosomes 1, 5 and 9 and to both ends of the remaining chromosomes. Scaffolds are named and listed based on their chromosomal assignments.

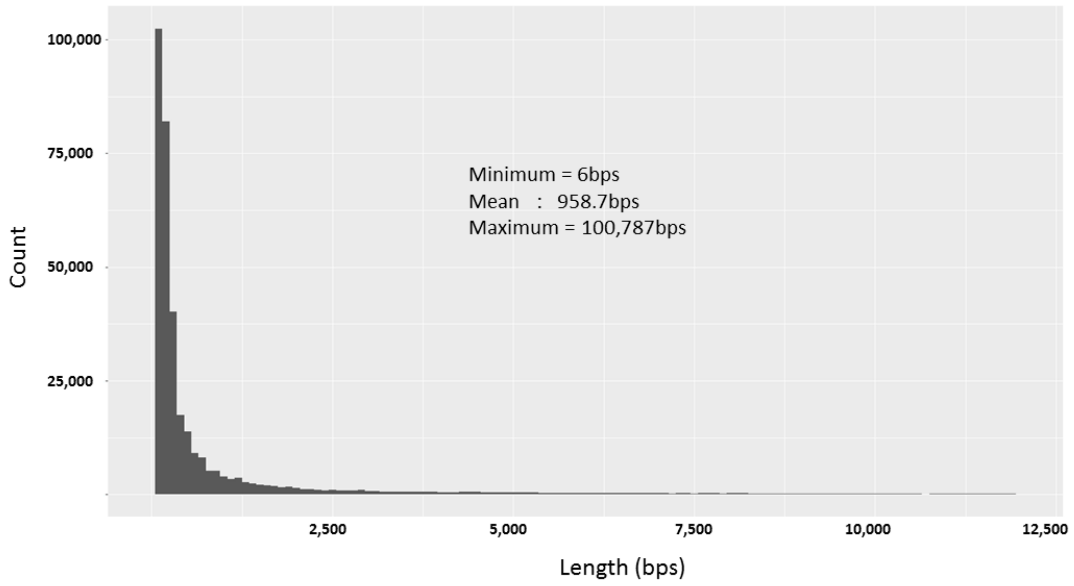


**Supplementary Figure 9: Determination of length of repeat regions *vs.* count in the Tx430 hybrid assembly.** Length of repeat regions were assessed by determining the length of masked regions after RepeatMasker screening.

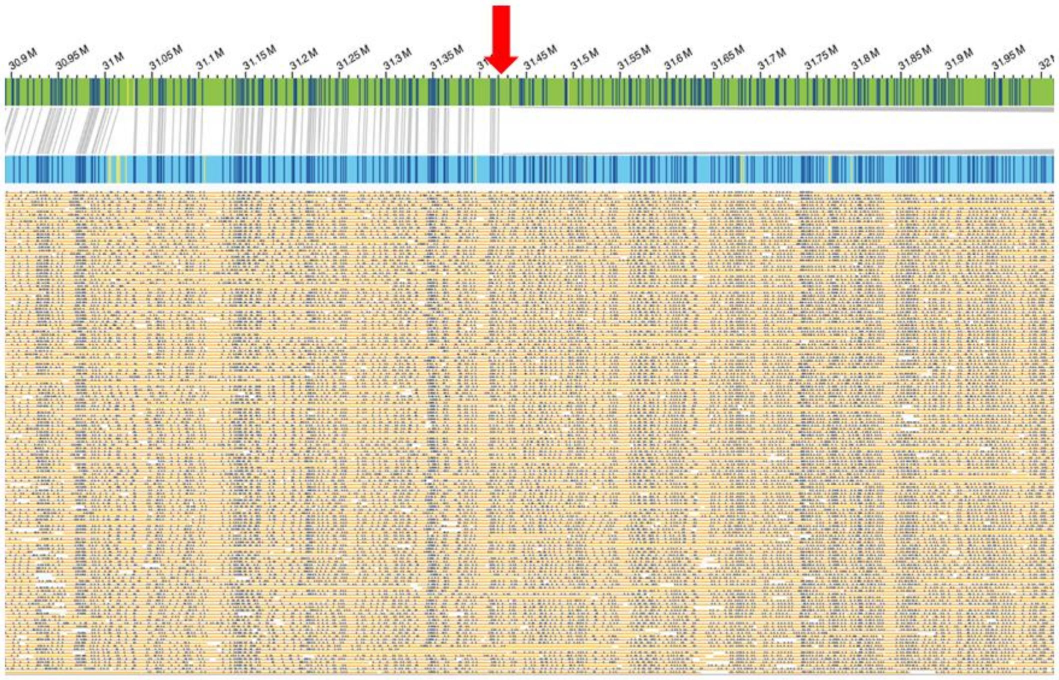

**Supplementary Figure 10: Close-up view of one chromosome 6 inversion breakpoint region in BTx623.** The *in-silico* map derived from the v3.0.1 BTx623 assembly is shown in green while the BTx623 DLS optical map is shown in blue. Overlapping individual Bionano molecules aligned to the DLS optical map and spanning the breakpoint area are shown below the map. The approximate location of the inversion breakpoint is marked by a red arrow.

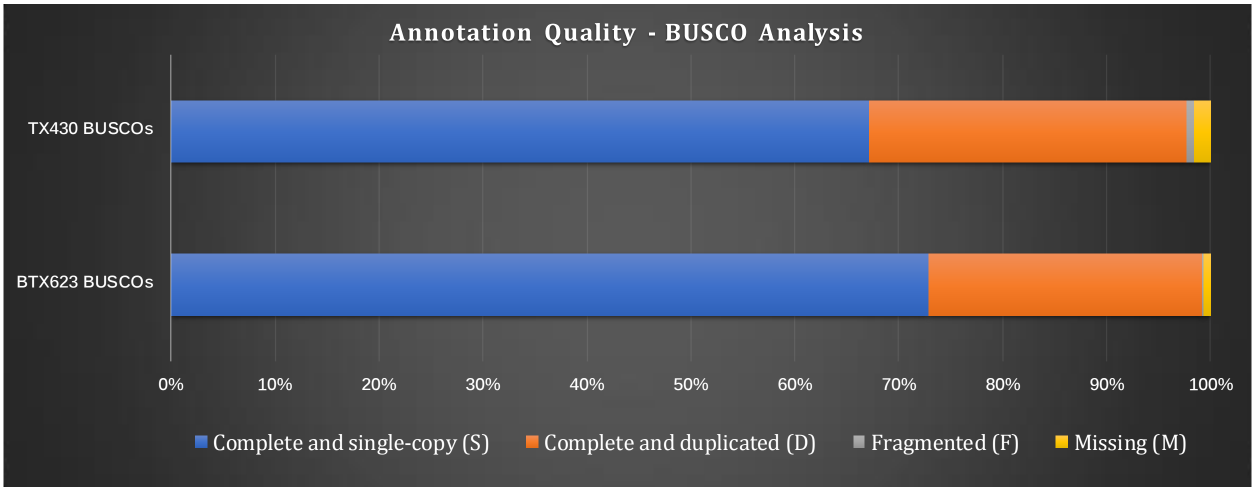


**Supplementary Figure 11: BUSCO analysis for annotation quality assessment.**

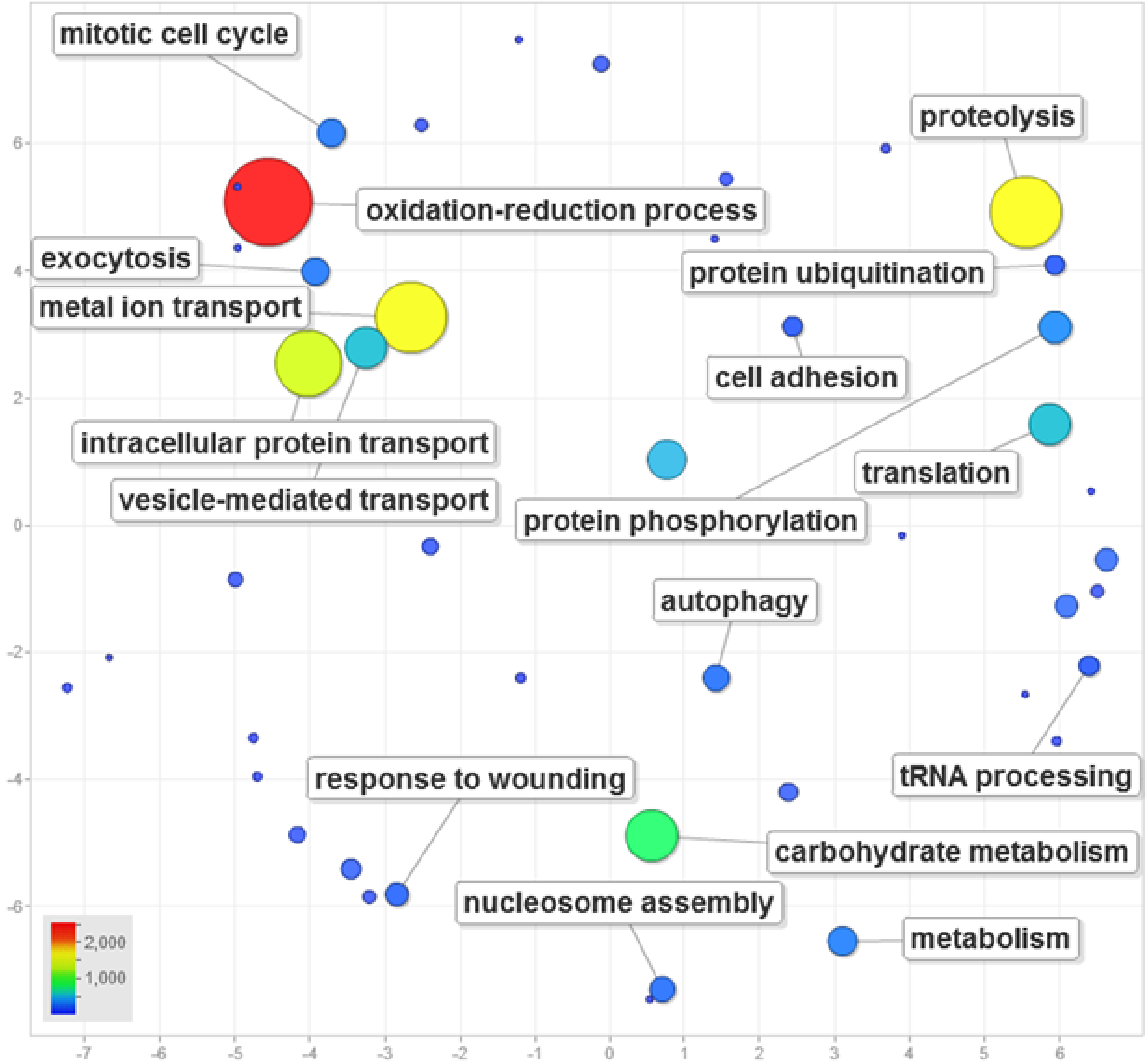


**Supplementary Figure 12: GO-term enrichment for biological process in 718 Tx430 proteins that are not homologous to any BTx623 protein.** Colors depict fraction of the proteins covered by a single GO term. Blue – low fraction, Red – higher fraction

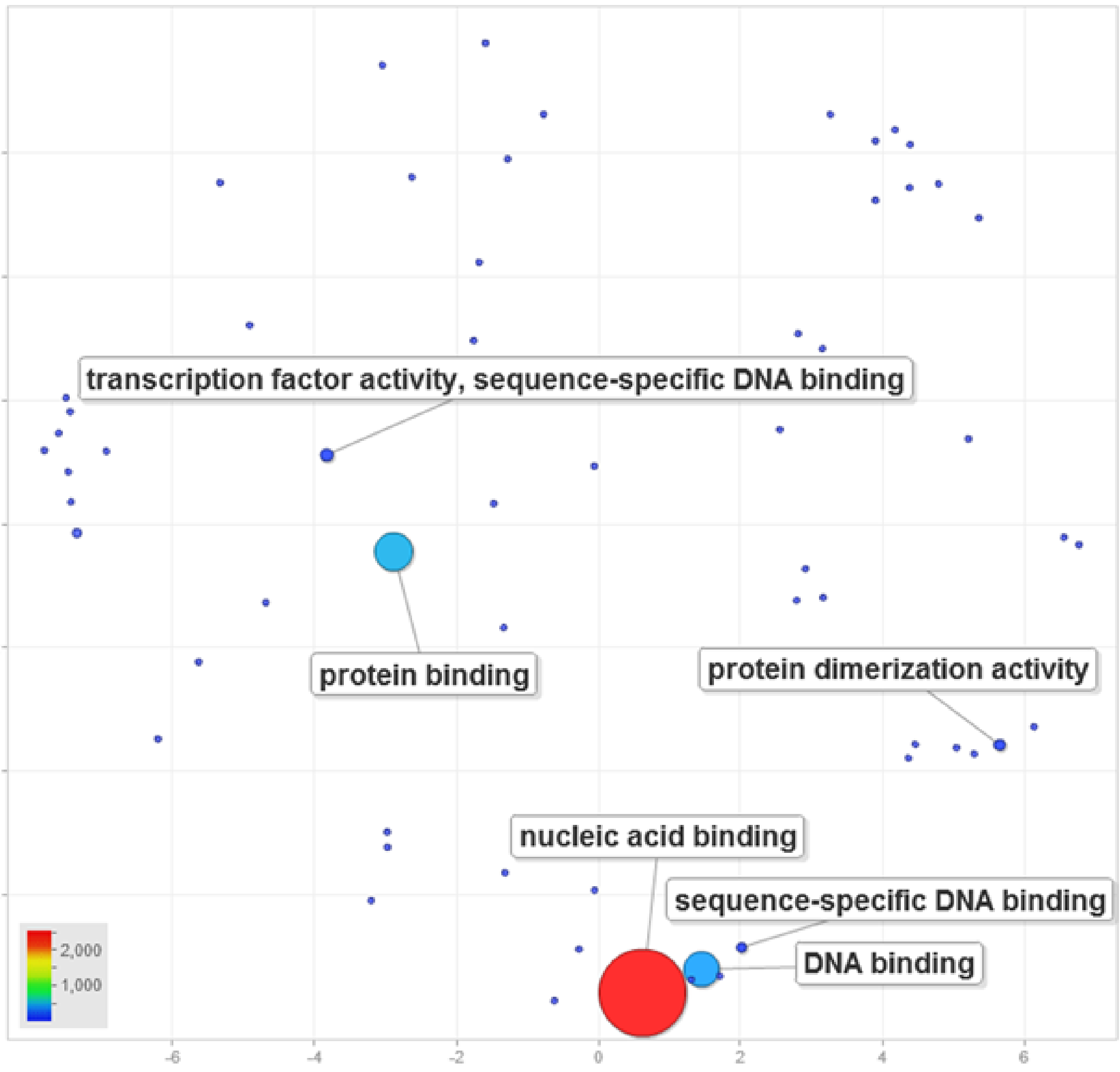


**Supplementary Figure 13: GO-term enrichment for molecular function in 718 TX430 proteins that are not homologous to any BTx623 protein.** Colors depict fraction of the proteins covered by a single GO term. Blue – low fraction, Red – higher fraction

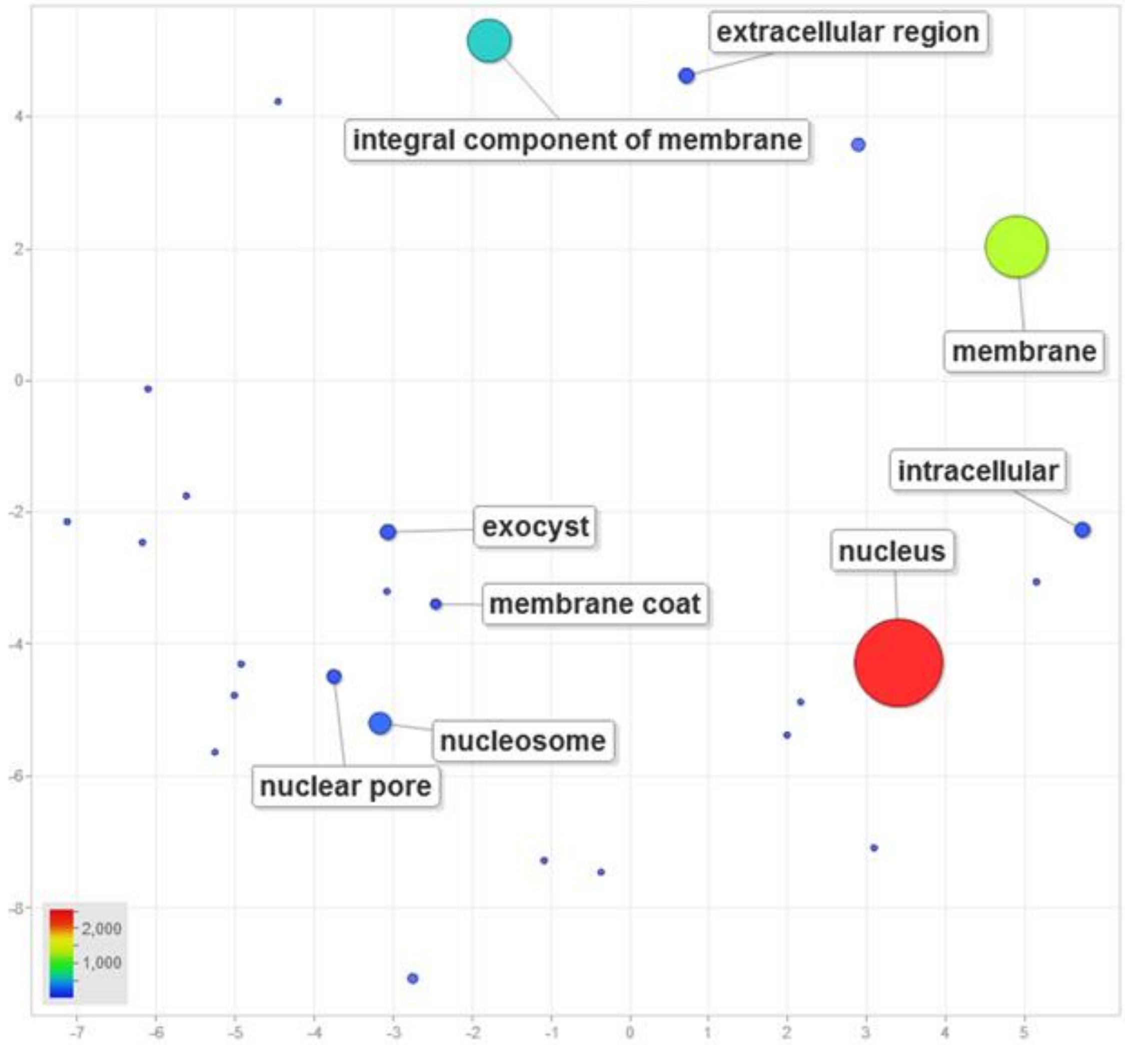


**Supplementary Figure 14: GO-term enrichment for cellular component in 718 Tx430 proteins that are not homologous to any BTx623 protein.** Colors depict fraction of the proteins covered by a single GO term. Blue – low fraction, Red – higher fraction

**Supplementary Table 1: Summary of sequencing run metrics on the MinION (SR = "Short Reads"; LR = "Long Reads")**

| MinION Runs | Number of reads | Number of bases | Mean read length (bps) | $N_{50}$ read length (bps) |
|---|---|---|---|---|
| SR1 | 622,099 | 4,644,995,691 | 7,467 | 8,542 |
| SR2 | 727,563 | 5,652,265,725 | 7,769 | 8,786 |
| SR3 | 123,706 | 980,098,593 | 7,923 | 9,613 |
| SR4 | 416,303 | 3,635,049,741 | 8,732 | 10,170 |
| SR5 | 578,142 | 4,536,332,937 | 7,846 | 8,968 |
| SR6 | 1,136,111 | 7,336,281,268 | 6,457 | 7,228 |
| SR7 | 1,059,720 | 6,740,546,727 | 6,361 | 7,124 |
| LR1 | 63,842 | 562,551,609 | 8,812 | 19,934 |
| LR2 | 24,401 | 231,228,623 | 9,476 | 21,447 |
| LR3 | 43,534 | 1,000,162,973 | 22,974 | 27,974 |
| LR4 | 4,440 | 114,776,296 | 25,851 | 38,037 |
| LR5 | 22,355 | 523,144,528 | 23,402 | 36,180 |
| LR6 | 41,228 | 862,021,880 | 20,909 | 34,713 |
| LR7 | 89,690 | 2,158,820,365 | 24,070 | 33,336 |
| LR8 | 135,416 | 2,950,632,392 | 21,879 | 32,525 |
| LR9 | 95,855 | 1,755,347,777 | 18,313 | 28,246 |
| LR10 | 157,482 | 3,167,308,207 | 20,112 | 30,954 |
| LR11 | 233,125 | 4,695,207,834 | 20,140 | 29,869 |
| LR12 | 152,273 | 1,562,164,979 | 10,259 | 15,639 |
| LR13 | 184,229 | 2,354,548,651 | 12,781 | 19,272 |
| LR14 | 303,600 | 4,101,399,523 | 13,509 | 20,198 |
| LR15 | 66,484 | 1,432,778,163 | 21,551 | 32,928 |
| LR16 | 96,917 | 2,308,041,410 | 23,815 | 38,180 |
| LR17 | 110,361 | 1,905,096,315 | 17,262 | 28,734 |
| LR18 | 44,016 | 820,398,292 | 18,639 | 32,656 |
| LR19 | 48,872 | 1,084,571,470 | 22,192 | 35,118 |

**Supplementary Table 2: Summary of assembly metrics ("LR" = Long reads only)**

| | CANU + SMARTdenovo (40X) | CANU + SMARTdenovo (60X) | CANU + SMARTdenovo (LR) |
|---|---|---|---|
| Number of Contigs | 1,366 | 1,059 | 740 |
| Total Length | 606,505,989 | 611,320,497 | 628,023,803 |
| Average Contig Length | 444,001 | 577,262 | 848,681 |
| Minimum Contig Length | 8,781 | 7,314 | 16,729 |
| Maximum Contig Length | 14,256,537 | 15,767,440 | 18,368,286 |
| $N_{25}$ Contig Length | 2,083,917 | 3,249,570 | 5,909,789 |
| $N_{50}$ Contig Length | 852,709 | 1,186,175 | 1,920,445 |
| $N_{75}$ Contig Length | 387,954 | 562,306 | 799,145 |

**Supplementary Table 3: Summary of correction and assembly input metrics**

| | Number of reads | Number of bases | Mean read length (bps) | $N_{50}$ read length (bps) |
|---|---|---|---|---|
| Total raw reads (>100bps) | 6,527,158 | 66,488,480,580 | 10,186 | 12,585 |
| Raw short reads (>2Kbps) | 4,397,189 | 32,417,762,086 | 7,372 | 8,067 |
| Raw long reads (>2Kbps) | 1,762,807 | 33,637,416,971 | 19,082 | 27,335 |
| Canu-corrected reads (>2Kbps) | 5,115,181 | 47,898,319,758 | 9,364 | 11,088 |
| Canu-corrected reads (>5Kbps) | 3,555,340 | 42,331,080,779 | 11,906 | 12,928 |

**Supplementary Table 4: Summary of the Tx430 DLS map generation and assembly**

| Input Molecules (filtered to >150Kbps): | | Molecules Aligned to the Reference: | |
|---|---|---|---|
| Total Number of >150Kbps molecules | 1,224,604 | Total Number of Molecules Aligned | 850,581 |
| Total Length of >150Kbps molecules (Mbps) | 340,107 | Fraction of Molecules Aligned | 0.695 |
| $N_{50}$ of >150Kbps molecules (Kbps) | 286.205 | Effective Coverage of Assembly (X) | 263.428 |
| Raw coverage of the reference (X) | 464.531 | Average Confidence | 36.1 |
| | | | |
| *De novo* Assembly: | | SV Summary: | |
| Genome Map Number | 79 | Deletion | 1,750 |
| Total Map Length/Reference Length | 0.982 | Duplication-Inverted | 120 |
| Genome Map $N_{50}$ (Mbps) | 33.773 | Insertion | 2,327 |
| Total Reference Length (Mbps) | 732.152 | Inversion Breakpoints | 52 |
| Total Number of Maps Aligned (Fraction) | 32 (0.41) | Translocation - Interchromosomal | 22 |
| | | Translocation - Intrachromosomal | 6 |

**Supplementary Table 5: Summary of the BTx623 DLS map generation and assembly**

| | | | |
|---|---|---|---|
| Input Molecules (filtered to >150Kbps): | | Molecules Aligned to the Reference: | |
| Total Number of >150Kbps molecules | 911,159 | Total Number of Molecules Aligned | 640,773 |
| Total Length of >150Kbps molecules (Mbps) | 256,923 | Fraction of Molecules Aligned | 0.703 |
| $N_{50}$ of >150Kbps molecules (Kbps) | 293.343 | Effective Coverage of Assembly (X) | 211.172 |
| Raw coverage of the reference (X) | 350.916 | Average Confidence | 35.3 |
| | | | |
| *De novo* Assembly: | | SV Summary: | |
| Genome Map Number | 44 | Deletion | 211 |
| Total Map Length/Reference Length | 0.988 | Insertion | 1,256 |
| Genome Map $N_{50}$ (Mbps) | 34.617 | Inversion Breakpoints | 30 |
| Total Reference Length (Mbps) | 732.152 | Translocation Breakpoints | 17 |
