# Supplementary material for: A chromosome-scale assembly of the sorghum genome using nanopore sequencing and optical mapping
Source: Nat Commun. 2018 Nov 19;9:4844. doi: 10.1038/s41467-018-07271-1 (PMC6242865; doi:10.1038/s41467-018-07271-1)
Supplement: Supplementary file 2 — Reporting Summary [file 41467_2018_7271_MOESM2_ESM.pdf]

nature research

Corresponding author(s): Stephane Deschamps and Haining Lin

Reporting Summary

Nature Research wishes to improve the reproducibility of the work that we publish. This form provides structure for consistency and transparency in reporting. For further information on Nature Research policies, see Authors & Referees and the Editorial Policy Checklist.

Statistical parameters

When statistical analyses are reported, confirm that the following items are present in the relevant location (e.g. figure legend, table legend, main text, or Methods section).

n/a	Confirmed	
☒	☐	The exact sample size (n) for each experimental group/condition, given as a discrete number and unit of measurement
☒	☐	An indication of whether measurements were taken from distinct samples or whether the same sample was measured repeatedly
☒	☐	The statistical test(s) used AND whether they are one- or two-sided<br>*Only common tests should be described solely by name; describe more complex techniques in the Methods section.*
☒	☐	A description of all covariates tested
☒	☐	A description of any assumptions or corrections, such as tests of normality and adjustment for multiple comparisons
☒	☐	A full description of the statistics including central tendency (e.g. means) or other basic estimates (e.g. regression coefficient) AND variation (e.g. standard deviation) or associated estimates of uncertainty (e.g. confidence intervals)
☒	☐	For null hypothesis testing, the test statistic (e.g. F, t, r) with confidence intervals, effect sizes, degrees of freedom and P value noted<br>*Give P values as exact values whenever suitable.*
☒	☐	For Bayesian analysis, information on the choice of priors and Markov chain Monte Carlo settings
☒	☐	For hierarchical and complex designs, identification of the appropriate level for tests and full reporting of outcomes
☒	☐	Estimates of effect sizes (e.g. Cohen's d, Pearson's r), indicating how they were calculated
☒	☐	Clearly defined error bars<br>*State explicitly what error bars represent (e.g. SD, SE, CI)*

Our web collection on statistics for biologists may be useful.

Software and code

Policy information about availability of computer code

Data collection	ONT Albacore Sequencing Pipeline Software (v2.0.1) - BioNano Genomics Access software suite (BioNano Genomics)
Data analysis	CANU (v1.6) - SMARTdenovo (v02-2018) - Pilon (v1.21) - RACON (v1.3.1) - RepeatMasker (v4.0.7) - Tandem Repeats Finder (v4.0.9) - BOWTIE2 (v2.3.4.3) -minimap2 (v2.10) - NUCmer 3.1 (MUMmer v3.9.4alpha) - MUMmerplot 3.5 (MUMmer 3.23 package) - Long Ranger ALIGN software (10X Genomics) - Supernova Assembler (10X Genomics) - Fgenesh (v7.2.2) - Augustus (v2.7) - SNAP (v2006-07-28) - GMAP (v03-25-2018) - PASA (v.2.2.2) - SPLAN (v2.1.3)

For manuscripts utilizing custom algorithms or software that are central to the research but not yet described in published literature, software must be made available to editors/reviewers upon request. We strongly encourage code deposition in a community repository (e.g. GitHub). See the Nature Research guidelines for submitting code & software for further information.

Data

Policy information about availability of data

All manuscripts must include a data availability statement. This statement should provide the following information, where applicable:

- Accession codes, unique identifiers, or web links for publicly available datasets
- A list of figures that have associated raw data
- A description of any restrictions on data availability

All data that support the findings of this study, including raw sequencing data, raw Bionano Genomics molecules and assembled maps, and the final polished assembly, have been deposited in the National Center for Biotechnology Information database under the BioProject accession number PRJNA472170 [https://www.ncbi.nlm.nih.gov/search/?term=PRJNA472170]. The Whole Genome Shotgun project has been deposited at DDBJ/ENA/GenBank under the accession QWKM00000000 [https://www.ncbi.nlm.nih.gov/search/?term=QWKM00000000]. The version described in this paper is version QWKM01000000 [https://www.ncbi.nlm.nih.gov/nuccore/QWKM01000000]. Raw sSequencing data have been deposited under SRA accession number SRP148505 [https://www.ncbi.nlm.nih.gov/search/?term=SRP148505]. All other relevant data are available from the corresponding authors on request. A reporting summary for this Article is available as a Supplementary Information file.

Field-specific reporting

Please select the best fit for your research. If you are not sure, read the appropriate sections before making your selection.

☒ Life sciences ☐ Behavioural & social sciences ☐ Ecological, evolutionary & environmental sciences

For a reference copy of the document with all sections, see nature.com/authors/policies/ReportingSummary-flat.pdf

Life sciences study design

All studies must disclose on these points even when the disclosure is negative.

Sample size	Genome sequencing and optical mapping generated single genome sequences and optical maps. As a result, the sample size for each is "1". Multiple plants were used to extract DNA for the study but, due to the nature of the work described here, this sampling size is not relevant to the conclusions reached in the study (natural variations in plant genome would translate into "mismatches" whose expected low frequency would be ignored due to the fact that sequences are corrected to generate one consensus sequence and one map or assembly per genome).
Data exclusions	No data were excluded from the analyses.
Replication	Genome sequencing and optical mapping were not replicated. The reproducibility of the experimental findings were ensured by the use of multiple independent techniques and technologies leading to "replicated" raw data information (such as DNA sequences all mapping to the same region with same sequences).
Randomization	Randomization was not relevant to this study. While plants selected for DNA extraction were selected "at random", no statistical analyses or evaluation of this process were possible or required.
Blinding	Blinding was not relevant to this study. No randomized or control trials of the data were performed or required.

Reporting for specific materials, systems and methods

Materials & experimental systems

n/a	Involved in the study
☒	☐ Unique biological materials
☒	☐ Antibodies
☒	☐ Eukaryotic cell lines
☒	☐ Palaeontology
☒	☐ Animals and other organisms
☒	☐ Human research participants

Methods

n/a	Involved in the study
☒	☐ ChIP-seq
☒	☐ Flow cytometry
☒	☐ MRI-based neuroimaging
